# Cold-related Florida manatee mortality in relation to air and water temperatures

**DOI:** 10.1371/journal.pone.0225048

**Published:** 2019-11-21

**Authors:** Stacie K. Hardy, Charles J. Deutsch, Tiffanie A. Cross, Martine de Wit, Jeffrey A. Hostetler

**Affiliations:** 1 Fish and Wildlife Research Institute, Florida Fish and Wildlife Conservation Commission, St. Petersburg, Florida, United States of America; 2 Fish and Wildlife Research Institute, Florida Fish and Wildlife Conservation Commission, Gainesville, Florida, United States of America; Hawaii Pacific University, UNITED STATES

## Abstract

Many tropical and subtropical species are sensitive to sudden temperature changes, especially drops in temperature. During winters 2009–2010 and 2010–2011, unusually cold temperatures occurred in many parts of Florida, USA, resulting in increased mortality of Florida manatees, sea turtles, fish, corals, and other species. The Florida manatee, in particular, is highly susceptible to cold stress and death when water temperatures drop below 20°C. We sought to characterize the magnitude and timing of reports of cold-related manatee carcasses in relation to fluctuations in water and air temperatures in central-east and central-west Florida during the six winters from 2008 to 2014. We used a generalized linear model to predict counts of manatee carcasses with a cold-related cause of death reported over 7-day bins in relation to various short-term (two weeks or less) and cumulative (incrementally summed from the start of the winter) heating-degree-day effects (HDD; < 20°C) and a categorical winter variable. Using water temperature data, the top-ranked model in both regions included a short-term temperature effect (14-day HDD sum) that preceded increases in reports of cold-related manatee carcasses by 7 days. Cumulative exposure to cold weather over the winter amplified effects on mortality in the central-east region. Quantifying the relationship between cold events and manatee mortality helps us prepare for rescue and salvage operations when extremely cold weather is forecast. This is especially important because anticipated loss or degradation of warm-water refuges due to human activities and sea level rise could potentially impact the manatee population in the future. These methods could also be applied to other species susceptible to cold-related mortality.

## Introduction

Many tropical and subtropical species have evolved in climates with a narrow temperature range and are particularly sensitive to extreme fluctuations in temperature [1]. During winter 2009–2010, Florida, United States of America experienced periods of severe cold, with temperatures throughout much of the state dropping to or below freezing. Sea turtles, coral reefs, invasive Burmese pythons, fish, and Florida manatees (*Trichechus manatus latirostris*) were among the most negatively affected [2–7]. Recent studies have investigated the effects of severity and timing of cold events on associated morbidity and mortality of sea turtles [2,8,9]. Although much work has been done to elucidate habitat use by manatees during winter and to describe warm-water habitats on which they rely [10–16], we were particularly interested in describing the timing between cold events and subsequent cold-related manatee mortality.

The Florida manatee is a threatened subtropical aquatic mammal found in the southeastern United States, primarily Florida [17]. A significant natural threat to Florida manatees is cold weather because their exceptionally low metabolic rate and poor insulation limit their physiological capacity to thermoregulate in cold water [18,19]. Prolonged exposure to cold water can result in cold stress syndrome (CSS) and death [20–23]. When water temperatures drop below 20°C, manatees migrate to warm-water habitats and sites that provide thermal shelter [18,24,25].

The number of cold-related manatee deaths in Florida varies across years depending on winter severity, spiking most drastically in winters 2009–2010 and 2010–2011 when record numbers of cold-related carcasses were documented (S1 Fig) and unusual mortality events were declared [5]. The U.S. Marine Mammal Protection Act defines an unusual mortality event (UME) as “a stranding that is unexpected; involves a significant die-off of any marine mammal population; and demands immediate response” [26]. The impact of these events on the manatee population is not yet fully understood, but estimated mortality rates of immature manatees, in particular calves, were substantially elevated [27]. During the winter 2009–2010 UME, 480 dead manatees were reported and verified statewide, of which 240 were calves; at least 252 of the 480 deaths were determined to have been caused by cold exposure [5].

Loss of warm-water habitat has long been identified in state and federal management plans as a threat to the long-term sustainability of the Florida manatee [28,29]. Continued warm-water habitat losses are anticipated over the coming decades due to declines in spring discharge and retirement of older technologies at power plants, which will reduce, if not eliminate, thermal discharge at many power plants currently used by manatees for warm-water refuge [10,27,30]. Operations at many coastal power plants will likely be adversely affected if sea level rises by the predicted 0.5–1.4 m above the 1990 level by the year 2100 [31]. Finally, many natural warm-water sites may be compromised due to climate change by intrusion of colder saltwater or reduced spring flow [32]. These changes will decrease the environmental carrying capacity for Florida manatees and, therefore, are expected to result in a smaller equilibrium population size and a slightly greater probability of quasi-extinction over the next 100 years [27].

Given the recent manatee die-offs from severe cold and the current and expected changes in warm-water availability, we sought to better understand the relationship between cold-related manatee mortality and air and water temperatures. Our objectives were (1) to identify the short-term and cumulative temperature effects most closely associated with manatee cold-related mortality in winter; (2) to determine the lag time between a temperature drop during a cold front and reports of manatees whose deaths were attributable to cold; and (3) to infer the heating-degree-day thresholds below which we can expect increased reports of cold-related mortality.

## Materials and methods

### Manatee carcass salvage and necropsy

Florida manatee carcasses were reported (by the public, research partners, or research staff) to a state-managed wildlife hotline, and most carcasses were transported for necropsy to the Marine Mammal Pathobiology Laboratory of the Florida Fish and Wildlife Conservation Commission’s Fish and Wildlife Research Institute [33]. The salvage, transportation, and necropsy of carcasses were conducted by authorized personnel in accordance with section 6 of the Endangered Species Act (implementing regulations 50 CFR 17.21(c) and 50 CFR 17.31(b)) and section 109(h) of the Marine Mammal Protection Act (implementing regulation 50 CFR 18.22). Badly decomposed carcasses were often necropsied in the field, and if a carcass was visually verified in the field but not recovered for a necropsy, it was assigned a cause of death as “verified: not recovered.” Necropsied carcasses were assigned one of the following specific causes of death based on significant findings during necropsy: human-related: watercraft collision; human-related: canal/lock; human-related: other; perinatal (young calf with total length ≤ 150 cm); natural: cold stress; natural: other (including red tide); undetermined: too decomposed; or undetermined: other. The condition of a carcass was assigned to one of four levels: fresh (no signs of decomposition); moderately decomposed; badly decomposed; and dried up or consisting mostly of loose bones. All cause of death and recovery data were stored in a relational database, double-verified after entry, and reviewed for consistency. Recovery locations were verified in ArcGIS (Esri, Redlands, CA) based on details provided in the recovery report.

Death from cold stress syndrome was determined by an evaluation of case history (i.e., likely cold exposure) and necropsy findings. External signs of cold exposure included epidermal bleaching and various types of dermal lesions (plaques, ulceration, pustules, and abscesses); internal signs of CSS included fat depletion, no gastrointestinal contents except presence of dry, hard feces, and secondary infection or inflammation (e.g., pneumonia or enteritis) [22]. During periods of extremely low temperatures, manatee carcasses with nonspecific findings, such as congestion (which suggests cardiovascular collapse) and drowning, were presumed to have died from cold shock and hypothermia [5], once we ruled out other causes of shock, such as brevetoxicosis.

Our dataset consisted of the number and report dates of carcasses with a cold-related cause of death from December through March for winters 2008–2009, 2009–2010, 2010–2011, 2011–2012, 2012–2013, and 2013–2014. We used the date the carcass was reported to the wildlife hotline to sum daily carcass counts into 7-day bins (starting December 1 each winter) to have sufficient count data without losing much temporal resolution. Decomposition state of the carcass was not considered when daily carcass counts were calculated, as we are not able to use condition to estimate when the manatee died. Carcasses assigned to the worst condition code (dried up or consisting of mostly loose bones) were excluded from our analyses, because they were too decomposed for their report date to be meaningful for analyses. Perinatal carcasses were excluded from our analyses because the physiological response to cold of young calves is probably not representative of older animals.

### Study area

**Fig 1:**
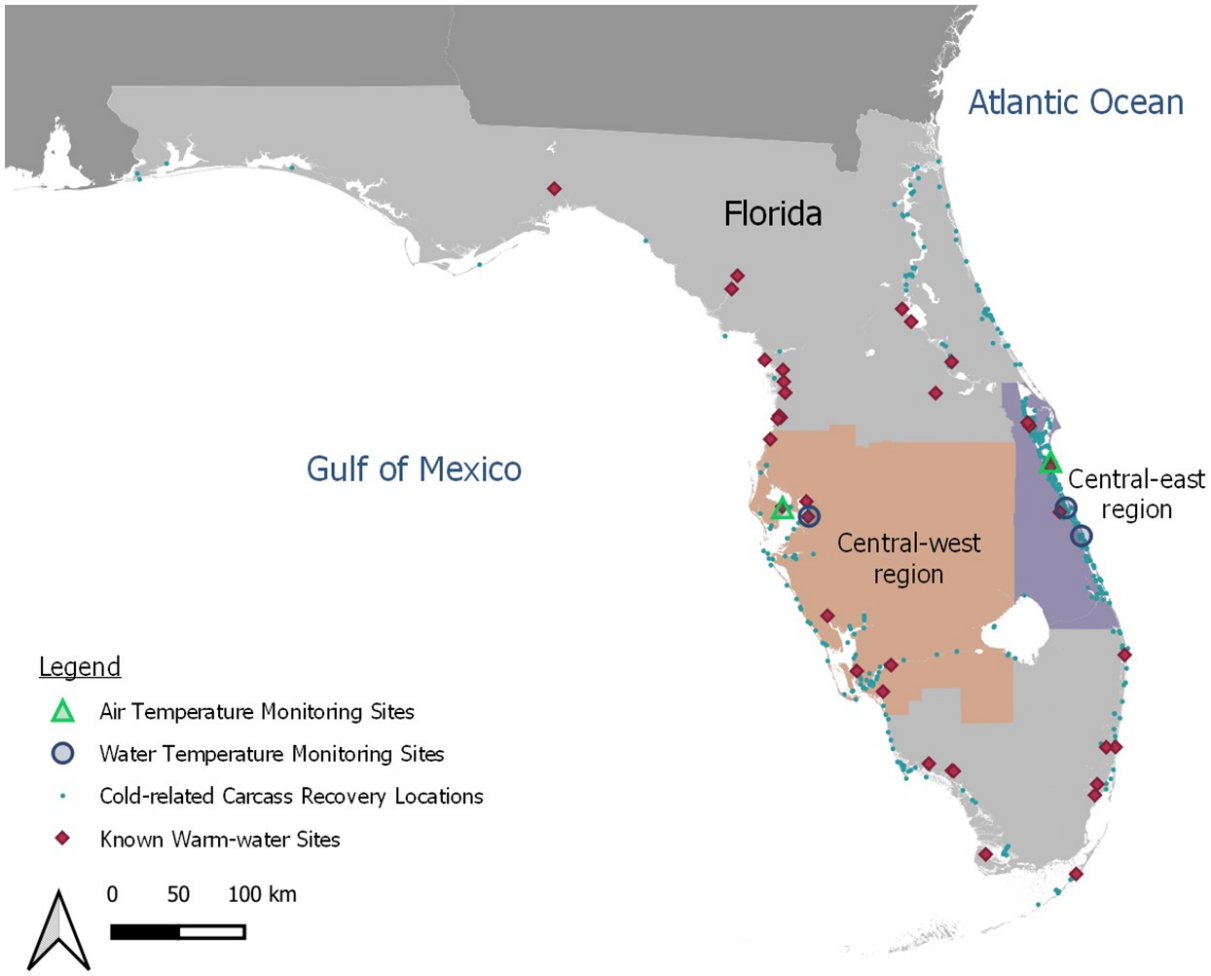
Extents of the central-east (CE) and central-west (CW) regions and locations of temperature monitoring sites. The CE and CW regions include coastal and inland water bodies through which manatees travel and in which carcasses have been found; the extents include the entirety of inland counties to ensure that all manatee carcasses are assigned a geographic region. The monitoring sites used for modeling cold-related manatee deaths are represented by blue circles (water temperature) and green triangles (air temperature). All cold-related manatee carcass recovery locations from winters 2008–2009 to 2013–2014 are depicted in the map as small blue circles, and the locations of known warm-water sites available to manatees are depicted as red diamonds.

After the 2009–2010 cold-related UME, we delineated six geographic regions for analyzing mortality data [5]. We assigned each carcass to one of these regions based on its recovery location and focused our analyses on the two regions of Florida—central-east (CE) and central-west (CW) (Fig 1)—that incurred the highest numbers of manatee deaths from cold stress during the winter 2009–2010 UME [5]. Given the high density of urban development in both regions, we assumed that the time between a manatee’s death and the reporting of its carcass to the wildlife hotline would have been similar, allowing us to compare model results between the two regions. We did not conduct any dedicated surveillance for carcasses in either region.

### Temperature monitoring and variable development

The Fish and Wildlife Research Institute’s Manatee Research Program monitored temperatures at selected warm-water manatee habitats and nearby ambient (i.e., non-warm-water) sites around Florida. We deployed temperature loggers (HOBO Water Temp Pro v2 Logger, Onset Computer Corp., Bourne, MA) to record air and water temperatures every 30 minutes from November 1 to March 31 every winter. Temperature data were reviewed for gross anomalies (e.g., due to water-temperature loggers being exposed to air or to logger malfunction) and imported into a relational database [34].

The standardization of temperature data collection was important for this analysis, so we selected temperature monitoring sites to provide representative mean daily temperatures for modeling cold-related manatee mortality in each region (Fig 1). We selected representative temperature monitoring sites based on the following criteria: completeness of data for winters 2008–2009 through 2013–2014; central location within the region in nearshore waters; and narrow daily temperature range. For water-temperature sites, we used temperature loggers deployed near the middle or bottom of the water column to limit temperature fluctuations due to cloud cover, direct sunlight, rain, or other external factors affecting the surface layer. These sites do not fully reflect the entire area’s temperature regime; localized areas may have warmer or cooler temperatures based on a number of factors, such as latitude, water depth, and water turnover.

For the CE region, the primary water temperature station was located in the southern Indian River at Vero Beach (Fig 1), with the logger fixed 0–0.25 m from the bottom in water 1.1–1.8 m deep (variation in deployment depth was due to differences in deployment among winters and variation in measured water depth was due to seasonal and tidal fluctuations). Data from this site were unavailable for winter 2012–2013, so a nearby site in the southern Indian River (25 km to the north) with a similar thermal regime was used for that winter; the logger was located 1.2 m from the bottom in water 2.0–2.7 m deep (variation in measured water depth was due to seasonal and tidal fluctuations).

For the CW region, the primary water temperature station was located in the Tampa Electric Company (TECO) Big Bend Power Station intake canal in Tampa Bay, with the logger fixed 1.1–3.9 m from the surface in water 5.1–8.5 m deep (variation in deployment depth was due to differences in deployment among winters and variation in measured water depth was due to seasonal and tidal fluctuations and slightly different locations; Fig 1). For air-temperature stations, we deployed temperature loggers in shaded locations to prevent direct sunlight from influencing recorded temperatures. Air temperature data were collected at a canal in Satellite Beach in the CE region and at Duke Energy Bartow Plant in St. Petersburg in the CW region (Fig 1).

Manatees are most susceptible to CSS when exposed to water temperatures below 20°C for an extended period [22], so we calculated a heating-degree-day (HDD) value for each site and day during the winter. HDD is a measurement used by the U.S. National Weather Service to describe how much energy is needed to warm a building when the mean daily outdoor temperature drops below a specific threshold; no energy is required to warm a building when the temperatures are above the threshold, so the HDD value defaults to zero. Because CSS is temperature-related and because we assume that manatees are not susceptible to CSS when temperatures exceed 20°C, we applied this measurement concept to our analysis and calculated HDD values for our air and water temperature data using a threshold of 20°C. For daily mean temperatures of 20°C or more, the HDD value is set to 0. For daily mean temperatures less than 20°C, the HDD value is 20 minus the daily mean temperature. Compared to mean daily temperatures, HDD better captured the effect of significant temperature drops because warmer days did not confound the results of the model.

**Fig 2:**
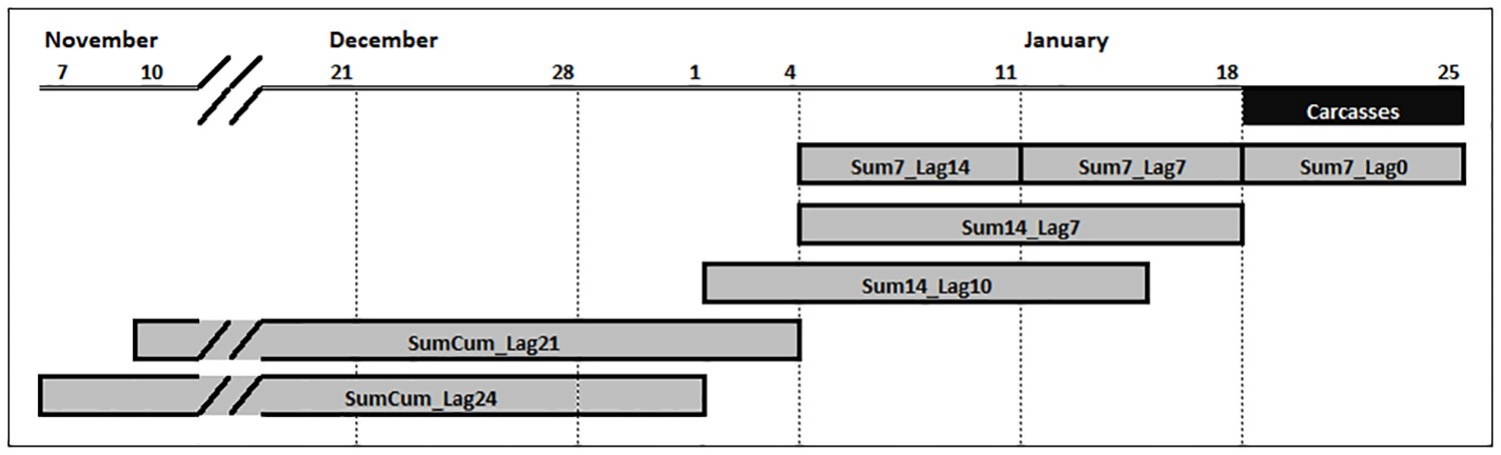
Visual representation of temperature covariates used in generalized linear models for analysis of cold-related manatee mortality. This figure displays the date range and overlap of temperature covariates relative to a 7-day carcass window from 19–25 January. The short-term temperature effects are named Sum#_Lag#, and the cumulative temperature effects are named SumCum_Lag# (where # indicates the number of days the data were summed or lagged).

We expected both short-term and cumulative temperatures from the start of the winter to influence the extent and timing of cold-related mortality. During the two cold UMEs, we observed a clear time lag between the sharp temperature drop associated with a strong cold front and an increase in the number of manatee carcasses reported [5]. This likely occurred because most manatees did not immediately succumb to CSS and because the carcasses were not immediately detected following the death of the manatee. To reflect these observations in our models, we developed short-term temperature effects to account for temperature changes over 7- or 14-day periods during or just prior to the 7-day carcass window and cumulative temperature effects to account for the cumulative effects of cold exposure on manatee condition from the start of winter to either 21 or 24 days before the 7-day carcass window (Fig 2). We selected 5 short-term temperature effects (HDD summed over 7 or 14 days, and lagged by 0, 7, 10, or 14 days) and 2 cumulative temperature effects (cumulative HDD summed since start of winter and lagged by 21 or 24 days) for use in our models (Fig 2).

When identifying temperature variables to test in our models, we ensured there was no temporal overlap between any of the temperature effects to minimize data duplication and multicollinearity. For example, for models including both short-term and cumulative temperature effects, we selected a cumulative temperature effect that ended *before* the short-term temperature effect started (Fig 2). We also tested the different combinations of temperature effects used in our models for multicollinearity using Pearson’s correlation coefficient. Pairs of variables that had a correlation coefficient > 0.7 or < -0.7 were deemed to be correlated (S1 Table). Two pairs of HDD variables were identified as correlated for the water data in the CW region: (1) 7-day sum, lagged by 0 days and 7-day sum, lagged by 7 days and (2) 7-day sum, lagged by 7 days and 7-day sum, lagged by 14 days. Since these correlated effects were limited to three models in the water data in the CW region, we tested all models for all regions to be able to compare the most parsimonious models across regions and temperature data sets.

### Modeling

Before testing the full suite of models, we tested several response variable distributions (Poisson, negative binomial, zero-inflated Poisson, and zero-inflated negative binomial) for both regions and temperature datasets using an interactive model between winter and a short-term temperature effect (14-day sum lagged by 10 days). We used c-hat ($\hat{c}$; sum of squared Pearson chi-square residuals divided by residual degrees of freedom) and chi-square goodness of fit (calculated on the same quantities) to evaluate response variable distributions (S2 Table) [35]. For those models that showed good fit ($\hat{c}<1.2$ and *p* > 0.05), the distribution with the lowest corrected Akaike information criterion (AICc) value was selected for use in analyses [36]. For the CE region, the negative binomial distribution provided the best fit for water and air temperature data (S2 Table; AICc model weights, relative to other distributions in model set, of 0.55 and 0.79, respectively). For the CW region, the Poisson distribution provided the best fit for water and air temperature data (S2 Table; relative model weights of 0.48 and 0.62, respectively).

We used a generalized linear model [35] to analyze variation in 7-day counts of manatee carcasses (whose death was attributed to cold) as a function of a winter categorical variable and short-term and cumulative temperature effects. We included the winter during which carcasses were reported (e.g. winter 2008–2009) as a categorical variable in select models, either as an additive term or an interactive term [37]. In all, we tested 61 models to identify the combination of variables that best explained the number of cold-related carcasses reported within a 7-day window in each region (S3 Table). We fit all models in R version 3.1 [38] using the MASS package [39] for four different data sets: one for each region using water temperature and one for each region using air temperature. We interpreted the most parsimonious model for each scenario tested as that having the lowest AICc value and the highest relative model weight. There are multiple competing methods for quantifying how well a model explains the data. We present a deviance pseudo-R^2^ value (*R*^*2*^_*DEV*_) for the most parsimonious model in each scenario, as this is often considered to be the most reliable pseudo-R^2^ for Poisson and negative binomial models [40,41]. We caution that this measure should not be compared across variable distributions or interpreted as being identical to true R^2^.

We modeled reports of cold-related manatee mortality knowing that there were factors affecting the temporal patterns of carcass reports that could have influenced our results. Carcass condition may be used as a rough index of the delay between a manatee’s death and the reporting of its carcass, but we do not have information about decomposition rates to estimate a death date based on the condition of the carcass at the time it was reported. We could not include terms for condition code in our models due to insufficient sample size, but we explored how the distribution of carcass condition codes changed after severe cold periods to better understand how that might have influenced model outcomes.

## Results

The six winters in our study varied from relatively mild to severely cold (S4 Table, S2 and S3 Figs). The number of manatee deaths from cold stress that we documented each winter ranged from 3 to 121 in the CE region and from 0 to 41 in the CW region (S4 Table). The numbers increased with winter severity (i.e., cumulative HDD over entire winter) in both regions (R^2^ was 0.898 and 0.910 for the CE and CW regions, respectively). More details on winter-specific patterns are presented in “S1 Text: Temperature patterns and cold-related mortality.”

### Model results using water temperature data in the CE region

**Fig 3:**
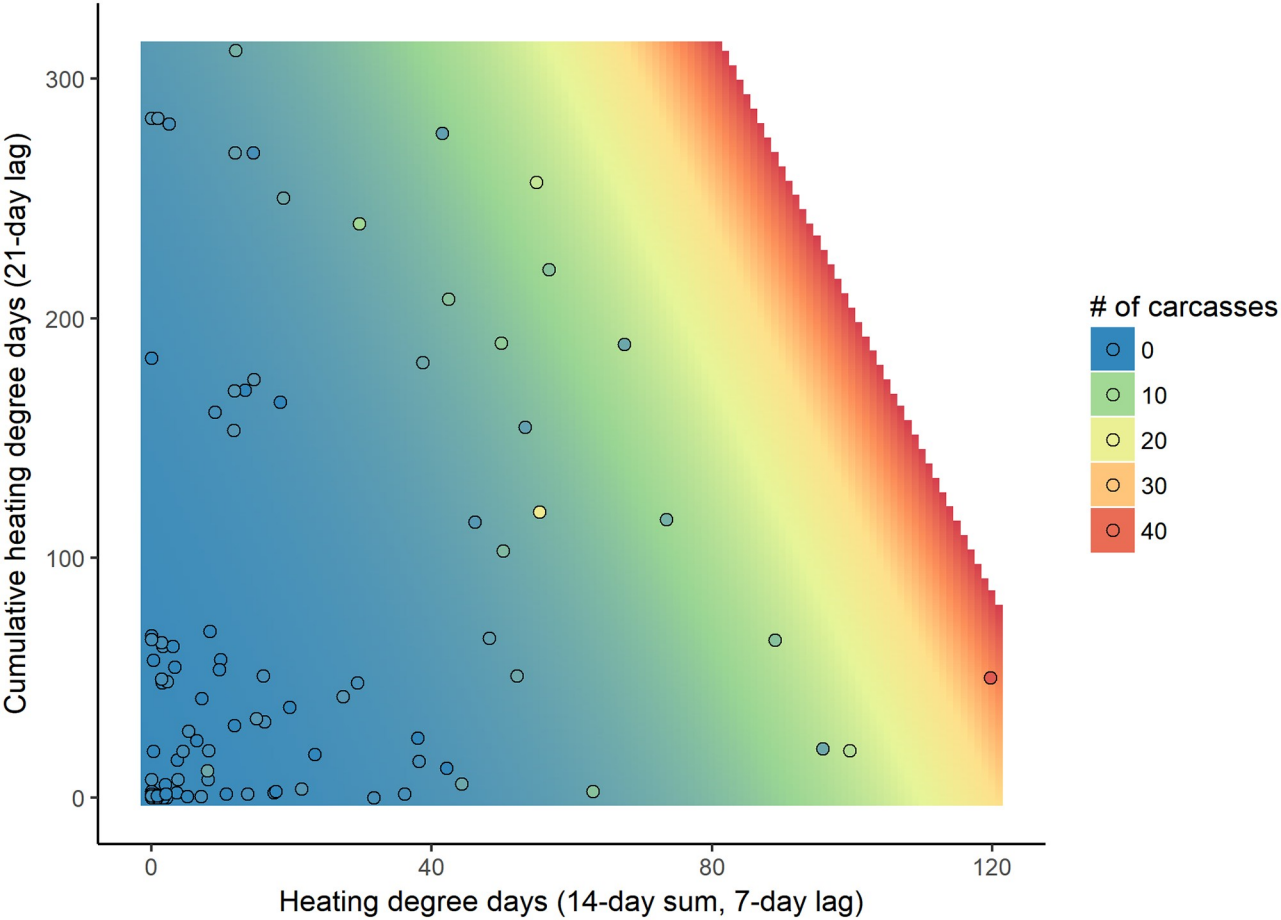
Predicted and observed 7-day counts of cold-related manatee carcasses reported in the central-east (CE) region using water temperature based on the short-term (14-day sum, lagged by 7 days; x-axis) and cumulative temperature effects (lagged by 21 days; y-axis) from the top-ranked model. The background color represents the predicted number of cold-related carcasses reported over 7 days, and the color of the circles represents the observed number of cold-related carcasses reported over 7 days. The scale for the number of carcasses is continuous (from blue to red), but for the ease of visualization, the legend displays the colors at intervals of 10 carcasses. We did not make predictions for the number of expected carcasses above the highest number reported during a 7-day period (area in white).

The additive model with a short-term temperature effect (14-day sum, lagged by 7 days) and a cumulative temperature effect (lagged by 21 days) best explained the number of cold-related deaths reported in the CE region using water temperature (Table 1). This model had a relative weight of 0.27 among the 61 models evaluated; the cumulative weight for the top 10 models was 0.94. *R*^*2*^_*DEV*_ for this model was 0.71. While winter was not included in the top-ranked model, it was included in the second model, which had a relative weight of 0.18. The predicted number of carcasses increased as the short-term temperature effect or the cumulative temperature effect increased; the short-term temperature effect was stronger (Fig 3). We confirmed that the short-term temperature effect was the stronger effect by standardizing our predictor variables (by subtracting the means and dividing by the standard deviation) and rerunning the models with the standardized versions of the predictor variables. The coefficients (± SE) for the standardized variables were 0.92 ± 0.08 for the short-term effect and 0.55 ± 0.09 for the cumulative effect.

**Table 1:** Top 10 models for 7-day counts of cold-related manatee carcasses reported in the central-east region based on water temperature.

Model	No. of parameters	ΔAICc	Weight
Sum14_Lag7 + SumCum_Lag21	4	0.000	0.265
Winter + Sum14_Lag7 + SumCum_Lag21	9	0.809	0.177
Sum7_Lag7 + Sum7_Lag14 + SumCum_Lag21	5	2.062	0.094
Sum7_Lag0 + Sum14_Lag7 + SumCum_Lag21	5	2.183	0.090
Sum14_Lag7 * SumCum_Lag21	5	2.203	0.088
Winter + Sum7_Lag7 + Sum7_Lag14 + SumCum_Lag21	10	3.127	0.055
Winter + Sum7_Lag0 + Sum14_Lag7 + SumCum_Lag21	10	3.135	0.055
Winter + Sum14_Lag7 * SumCum_Lag21	10	3.162	0.054
Winter + Sum14_Lag10 + SumCum_Lag24	9	4.148	0.033
Sum7_Lag0 + Sum7_Lag7 + Sum7_Lag14 + SumCum_Lag21	6	4.218	0.032

Reports of cold-related manatee carcasses were modeled using a negative binomial generalized linear model, and models were ranked using the AICc value. Temperature variables used in the models are described in Fig 2. Full model results are presented in S5 Table.

### Model results using air temperature data in the CE region

**Fig 4:**
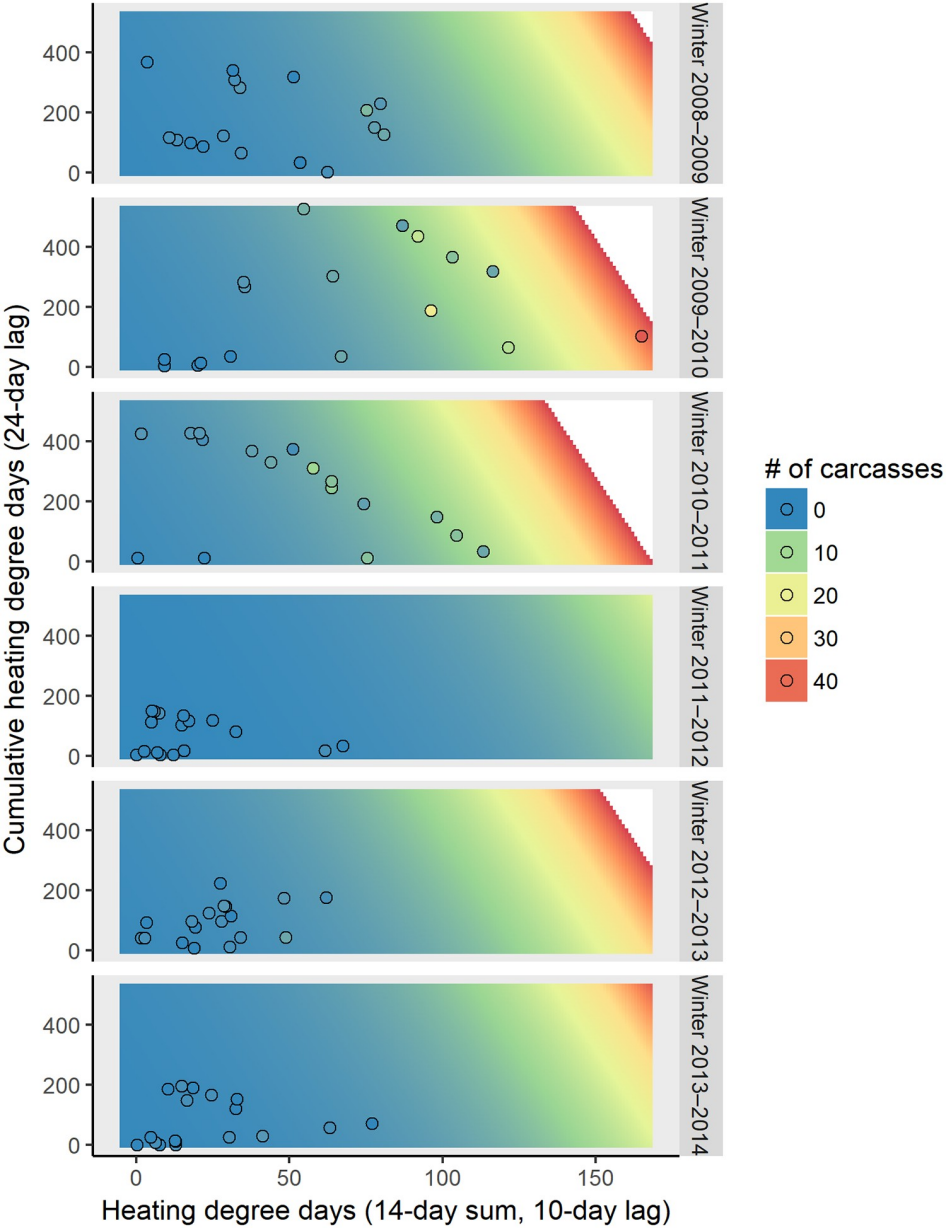
The predicted 7-day counts of cold-related manatee carcasses reported in the central-east region using air temperature based on the winter (each panel), short-term (14-day sum, lagged by 10 days; x-axis) and cumulative temperature effects (lagged by 24 days; y-axis) from the top-ranked model. Each panel represents the observed (circles) and predicted (colored area) number of cold-related carcasses reported over 7 days for each winter. The background color represents the predicted number of cold-related carcasses reported over 7 days, and the color of the circles represent the observed number of cold-related carcasses reported over 7 days. The scale for the number of carcasses is continuous (from blue to red), but for the ease of visualization, the legend displays the colors at intervals of 10 carcasses. We did not make predictions for the number of expected carcasses above the highest number reported during a 7-day period (area in white).

The model that best explained the number of cold-related carcasses reported in the CE region using air temperature consisted of an additive relationship between winter, a short-term temperature effect (14 day-sum, lagged by 10 days), and a cumulative temperature effect (lagged by 24 days) (Table 2). This model had a relative weight of 0.42 among the 61 models evaluated and an *R*^*2*^_*DEV*_ value of 0.74; the cumulative weights were 0.76 and 0.995 for the top 3 and top 10 models, respectively. As the value of the short-term temperature effect or the cumulative temperature effect increased, the predicted number of carcasses also increased (Fig 4). The predicted number of cold-related carcasses was highest in winters of 2009–2010 and 2010–2011 (when the greatest numbers of cold-related carcasses were reported) and lowest in winter 2011–2012 (when the fewest cold-related carcasses were reported).

**Table 2:** Top 10 models for 7-day counts of cold-related manatee carcasses reported in the central-east region based on air temperature.

Model	No. of parameters	ΔAICc	Weight
Winter + Sum14_Lag10 + SumCum_Lag24	9	0.000	0.419
Winter + Sum14_Lag10 * SumCum_Lag24	10	1.466	0.201
Sum14_Lag10 * SumCum_Lag24	5	2.207	0.139
Winter + Sum14_Lag10	8	2.517	0.119
Sum14_Lag10 + SumCum_Lag24	4	2.582	0.115
Winter * Sum14_Lag10	13	10.429	0.002
Winter + Sum7_Lag7 + Sum7_Lag14 + SumCum_Lag21	10	13.533	0.000
Sum7_Lag7 + Sum7_Lag14 + SumCum_Lag21	5	14.174	0.000
Winter + Sum14_Lag7 + SumCum_Lag21	9	14.440	0.000
Sum14_Lag7 + SumCum_Lag21	4	14.624	0.000

Reports of cold-related carcasses were modeled using a negative binomial generalized linear model, and models were ranked using the AICc value. Temperature variables used in the models are described in Fig 2. Full model results are presented in S6 Table.

### Model results using water temperature data in the CW region

**Fig 5:**
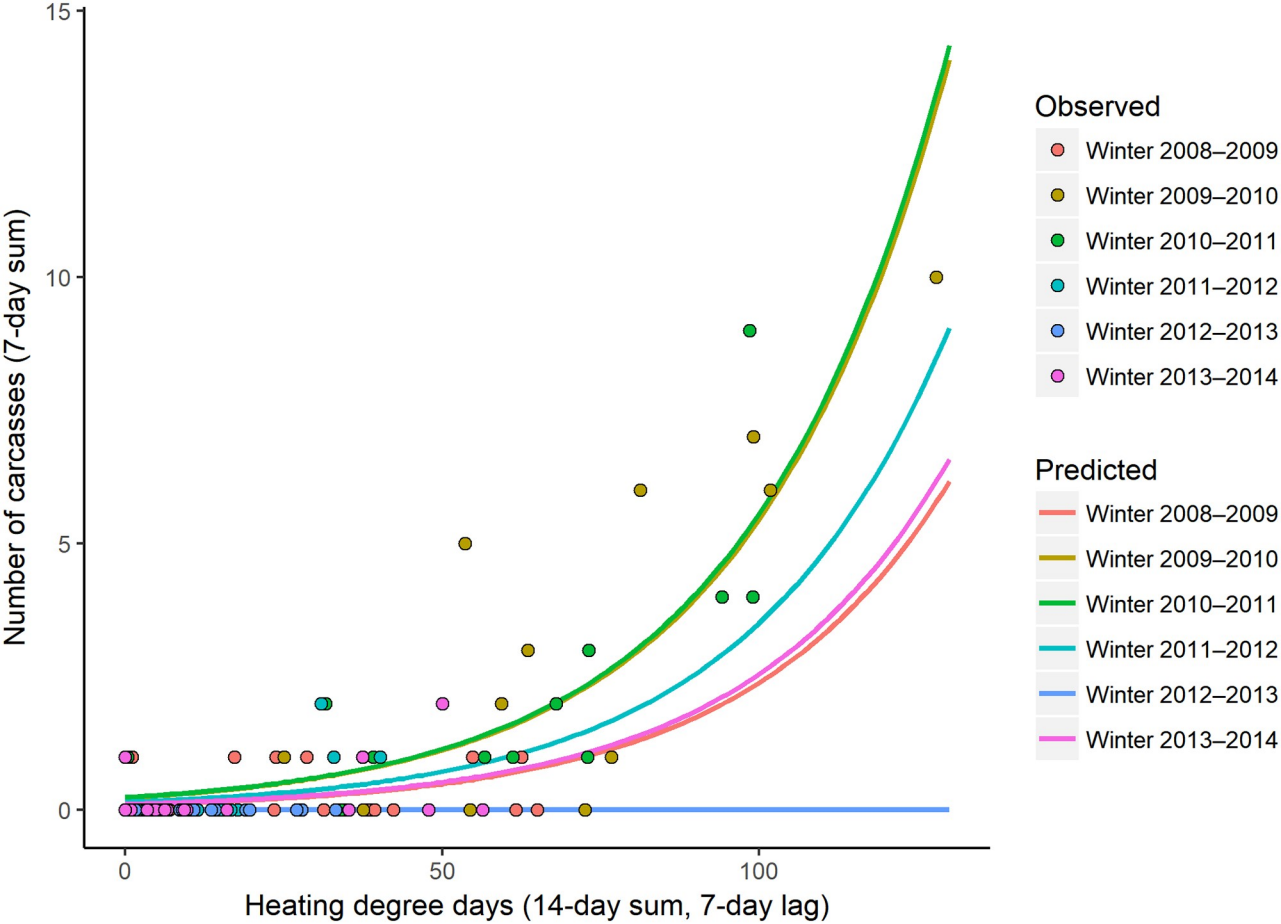
The predicted 7-day counts of cold-related manatee carcasses reported in the central-west region using water temperature based on the short-term temperature effect (14-day sum, lagged by 7 days; x-axis) by winter from the top-ranked model. Each line represents a different winter, and the circles represent the observed numbers of cold-related carcasses reported over 7 days color-coded by winter. Note that the prediction lines for winters 2009–2010 and 2010–2011 are nearly identical.

The model with an additive relationship between winter and a short-term temperature effect (14-day sum, lagged by 7 days) best explained the number of cold-related carcasses reported in the CW region using water temperature data (Table 3). This model had a relative weight of only 0.15 among the 61 models evaluated and an *R*^*2*^_*DEV*_ value of 0.71; the cumulative weight for the top 10 models was 0.72. The third-best model for the CW region was the same as that for the CE region: an additive relationship between a short-term temperature effect (14-day sum, lagged by 7 days) and a cumulative temperature effect (lagged by 21 days); this model had a relative weight of 0.09. More cold-related carcasses were predicted as the short-term temperature effect increased, and the highest number of carcasses was predicted in winters 2009–2010 and 2010–2011 (Fig 5).

**Table 3:** Top 10 models for 7-day counts of cold-related manatee carcasses reported in the central-west region based on water temperature.

Model	No. of parameters	ΔAICc	Weight
Winter + Sum14_Lag7	7	0.000	0.145
Sum14_Lag7 * SumCum_Lag21	4	0.804	0.097
Sum14_Lag7 + SumCum_Lag21	3	1.007	0.088
Sum7_Lag0 + Sum14_Lag7 + SumCum_Lag21	4	1.489	0.069
Sum7_Lag7 * Sum7_Lag14	4	1.592	0.065
Sum14_Lag7	2	1.832	0.058
Winter + Sum14_Lag7 + SumCum_Lag21	8	2.049	0.052
Sum7_Lag7 + Sum7_Lag14 + SumCum_Lag21	4	2.119	0.050
Winter + Sum7_Lag7 + Sum7_Lag14	8	2.157	0.049
Winter + Sum7_Lag0 + Sum14_Lag7	8	2.338	0.045

Reports of cold-related carcasses were modeled using a Poisson generalized linear model, and models were ranked using the AICc value. Temperature variables used in the models are described in Fig 2. Full model results are presented in S7 Table.

### Model results using air temperature data in the CW region

**Fig 6:**
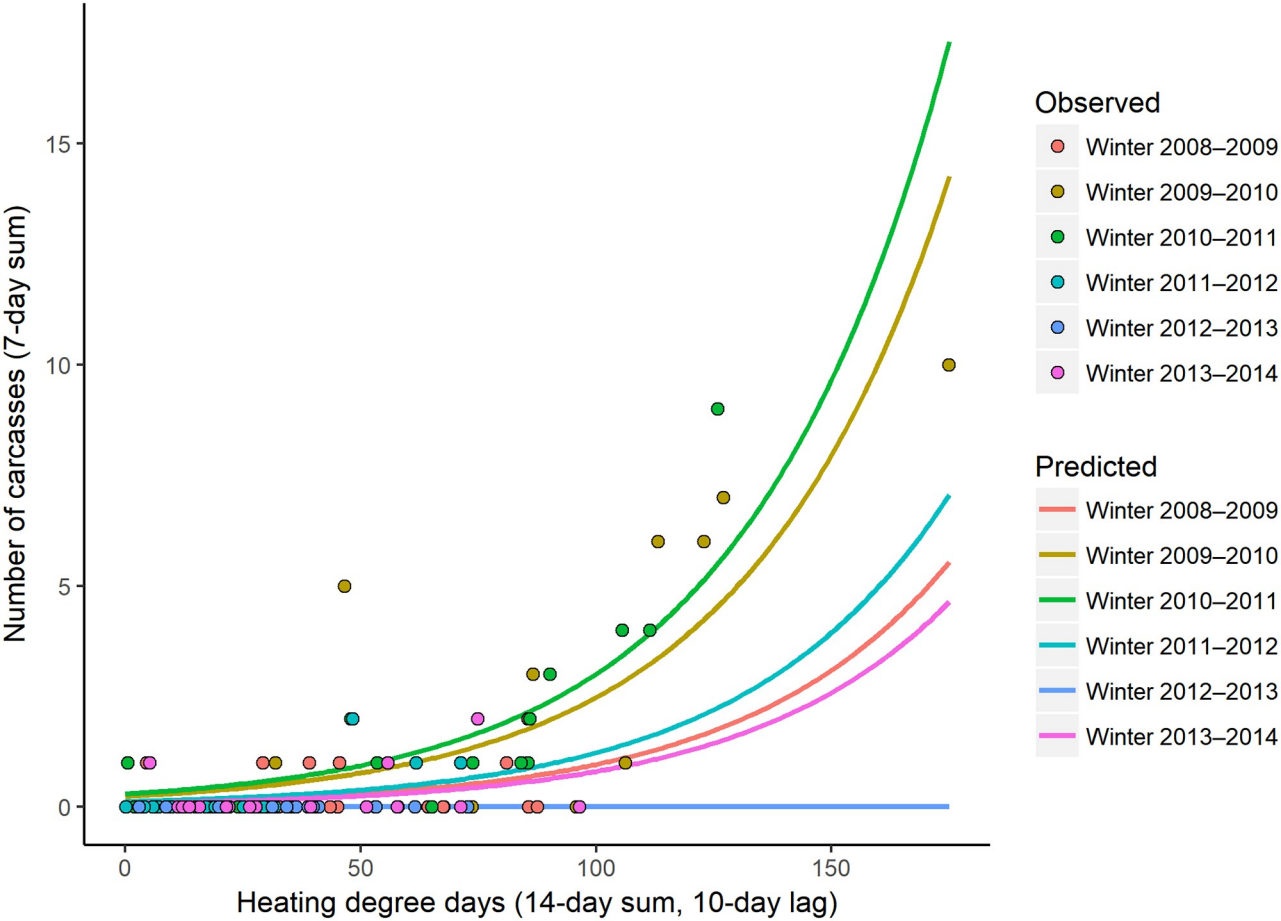
The predicted 7-day counts of cold-related manatee carcasses reported in the central-west region using air temperature based on the short-term temperature effect (14-day sum, lagged by 10 days; x-axis) by winter from the top-ranked model. Each line represents a different winter, and the circles represent the observed numbers of cold-related carcasses reported over 7 days, color-coded by winter.

When modeling CW cold-related mortality using air temperature data, the model that best explained the number of cold-related carcasses was an additive relationship between the winter categorical variable and a short-term temperature effect (14-day sum, lagged by 10 days) (Table 4). This model had a relative weight of 0.53 among the 61 models evaluated and an *R*^*2*^_*DEV*_ value of 0.68; the cumulative weight for the top 10 models was 0.98. Similar to what was seen in the model results for the CW region using water temperature, as the short-term temperature effect increased, the number of cold-related carcasses increased (Fig 6).

**Table 4:** Top 10 models for 7-day counts of cold-related manatee carcasses reported in the central-west region based on air temperature.

Model	No. of parameters	ΔAICc	Weight
Winter + Sum14_Lag10	7	0.000	0.530
Winter + Sum14_Lag10 + SumCum_Lag24	8	2.339	0.165
Winter * Sum14_Lag10	12	2.871	0.126
Winter + Sum14_Lag10 * SumCum_Lag24	9	3.196	0.107
Winter + Sum7_Lag7 + Sum7_Lag14	8	6.715	0.018
Winter + Sum7_Lag7 * Sum7_Lag14	9	8.411	0.008
Winter + Sum7_Lag0 * Sum7_Lag14	9	8.526	0.007
Winter + Sum7_Lag0 + Sum7_Lag7 + Sum7_Lag14	9	8.745	0.007
Winter + Sum7_Lag14	7	8.838	0.006
Winter + Sum7_Lag7 + Sum7_Lag14 + SumCum_Lag21	9	9.004	0.006

Reports of cold-related carcasses were modeled using a Poisson generalized model, and models were ranked using the AICc value. Temperature variables used in the models are described in Fig 2. Full model results are presented in S8 Table.

### Timing of cold-related manatee mortality by carcass condition

**Fig 7:**
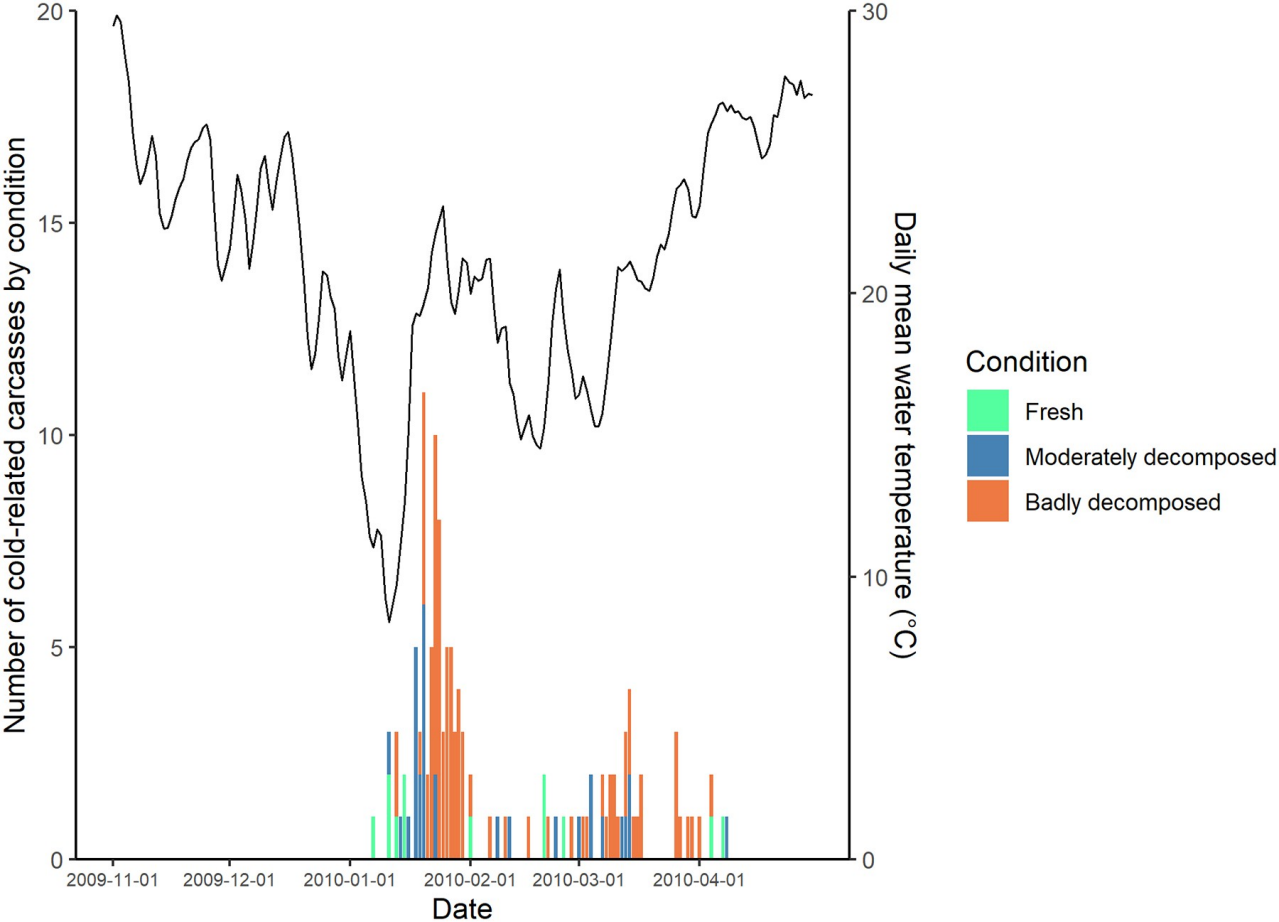
Number of cold-related manatee carcasses reported by day and condition code in the central-east (CE) region during winter 2009–2010. Mean daily water temperature from the water monitoring site in Indian River at Vero Beach (see Fig 1) is plotted in black. Note that the scales on the y-axis for carcass counts differ between Figs 7 and 8.

**Fig 8:**
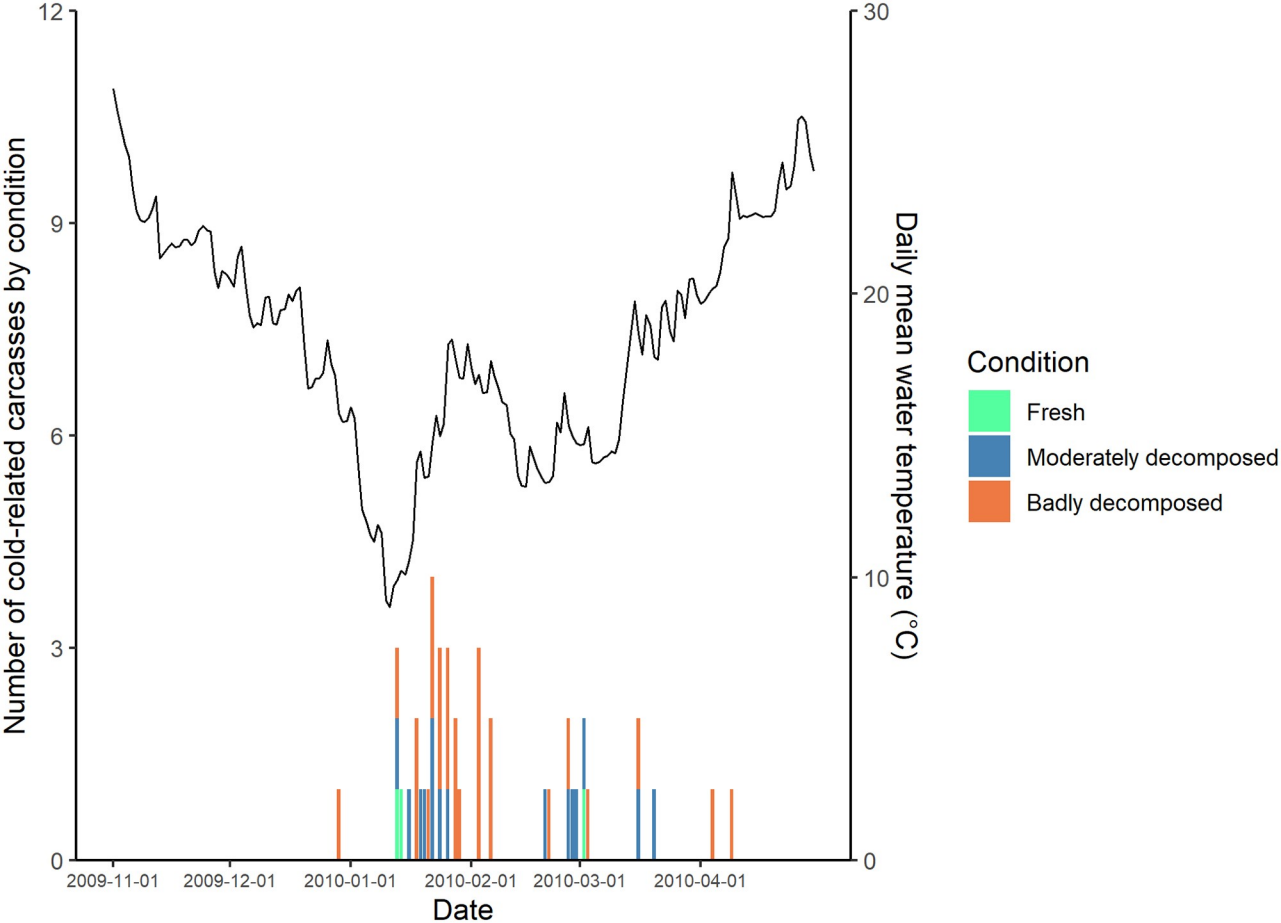
Number of cold-related manatee carcasses reported by day and condition code in the central-west (CW) region during winter 2009–2010. Mean daily water temperature from the TECO Big Bend Power Station intake (see Fig 1) is plotted in black. Note that the scales on the y-axis for carcass counts differ between Figs 7 and 8.

When we examined the carcass condition of manatees that had died from cold stress over the course of winter 2009–10, fresher carcasses were recovered sooner after the passage of strong cold fronts, followed progressively by more decomposed carcasses (Figs 7 and 8); this was most notable in the CE region (Fig 7).

## Discussion

### Relationship between cold-related mortality and temperature

We describe how the timing and magnitude of reports of cold-related manatee mortality are related to winter temperature patterns. Both short-term and cumulative temperature effects strongly influenced the number of cold-related manatee carcasses reported during 7-day periods in winter. When using water temperature data, the same short-term temperature effect (14-day HDD sum lagged to the carcass data by 7 days) was selected in the most parsimonious model for both regions; the period of this temperature effect begins 14 days before the start of the carcass period (Fig 2). The top-ranked models in both regions also included a longer-term effect: cumulative HDD lagged to the carcass data by 21 days in the CE region and a winter categorical effect in the CW region (Tables 1 and 3). When using air temperature data, the top-ranked model for both regions included a short-term temperature effect (14-day HDD sum lagged to the carcasses by 10 days) and an additive winter categorical effect. The top-ranked model for the CE region also included an additive cumulative air temperature effect lagged to the carcass data by 24 days (Tables 2 and 4). Based on AICc values, the water temperature-based models generally predicted the number of cold-related carcasses better than the models based on air temperature (S5–S8 Tables).

The short-term effects selected in the top-ranked models were similar whether water or air temperatures were used. This supports a clear relationship between cold-related manatee deaths and short-term temperature declines below 20°C. The main difference between the top water and air temperature models was the lag time between the arrival of cold weather (i.e. increased HDD values) and reports of cold-related carcasses: 7 days when using water temperature and 10 days when using air temperature. Water temperatures change more slowly than air temperatures, so it is expected that the time interval between water temperature declines and cold-related mortality is shorter than that between air temperature declines and cold-related mortality.

Although the severity of a given cold event has the most important effect on cold-related manatee mortality, cumulative exposure to cold during the winter also plays a role. The number of cold-related carcasses predicted after a single cold event was less during an otherwise relatively mild winter (low cumulative HDD) than during a colder winter (high cumulative HDD). For example, if the short-term HDD value in the CE region is 75 and water temperatures have not dropped below 20°C earlier in the winter (i.e., cumulative HDD = 0), approximately 5 cold-related carcasses are expected to be reported; if it has been a severely cold winter (i.e., cumulative HDD > 280), more than 25 carcasses are expected to be reported (Fig 3). This suggests that manatee health and survival become progressively compromised with continued exposure to cold water, which is consistent with published findings on the pathology of manatee CSS [22,23].

More cold-related manatee carcasses were observed (S4 Table) and predicted in the CE region than in the CW region. With a short-term HDD value of 100 using water temperatures, 12–40 carcasses are predicted in the CE region (depending on cumulative HDD) and 0–6 carcasses are predicted in the CW region (depending on winter) (Figs 3 and 5). We do not interpret these results to indicate that manatees tolerate cold temperatures differently in the two regions; instead, the difference reflects, in part, a larger regional abundance of manatees on the east coast of Florida [42]. Additionally, the number, types, and distribution of warm-water refuges vary between the two regions, which may differentially affect regional carcass counts: warm-water habitat is available throughout much of the CW region (8 known warm-water sites), whereas known warm-water habitat is limited to the northern half of the CE region (4–5 known warm-water sites during our study period) (Fig 1). The counts of carcasses will vary over time based on regional manatee abundance and other factors; it is the identification of key temperature covariates and their documented relationships with cold-related mortality that is the most important contribution of this study.

### Time lag between cold events and cold-related carcasses

The temporal relationship between temperature declines during the passage of cold fronts and cold-related manatee mortality is especially useful for on-the-ground response and carcass salvage efforts. Stranding personnel should be prepared to receive more reports of carcasses starting 1–2 weeks after water temperatures either drop below the 20°C threshold with one significant cold front or average below 20°C for two or more weeks. This also suggests that cases of live manatees with CSS can be expected within days after a 14-day period of water temperatures that average below 20°C.

The observed temporal lag between sharp drops in temperature and increased cold-related deaths results from at least two processes: biological factors and detection. First, the biological process representing time from cold exposure to death varies with the severity and duration of cold. Time to death can be short in cases of acute hypothermia or it can be protracted with the development of chronic CSS. Other variables include the size, health, and condition of the animal and the stability and warmth of water at aggregation sites in the region at the time of the cold spell [5]. The second process covers the time from death to detection. A carcass typically needs to be stranded onshore or floating to be available for detection, and detection probability can vary within a region based on distribution of human activity. Once detected, a carcass must be reported to authorities, which depends on the public’s willingness to report the sighting and knowledge of how to do so. The sum of these processes can range from a matter of hours to weeks, which is partly why we evaluated cold-related mortality over 7-day periods rather than daily. Although the top-ranked models for each region were not identical, they included the same short-term temperature effect, which suggests similarity in the reporting of carcasses for both regions and is consistent with the similar levels of development and human activity in the coastal and inland waters of these regions.

Based on our exploratory examination of carcass condition in winter 2009–2010, we found that, in general, recovered carcasses were progressively more decomposed as more time had elapsed since passage of a strong cold front (Figs 7 and 8). This suggests there may be more synchronicity of manatees succumbing to CSS than our data showed or than the models could predict. Although we are not able to predict exactly when manatees will die relative to the timing of temperature changes, understanding the thermal patterns that lead to increased reports of carcasses allows us to better prepare staff for increased salvage work and efforts to identify and, ideally, rescue cold-stressed animals.

### Heating-degree-day threshold effect

We identified heating-degree-day thresholds at which we might expect to see elevated numbers of carcasses reported over a 7-day period. Given present abundance, at least 10 cold-related carcasses would be expected to be reported in the CE region one week after the short-term HDD reaches 90 based on water temperatures, regardless of the cumulative temperature effect (Fig 3). This corresponds to a two-week period with water temperatures remaining below 20°C and averaging 13.6°C. In such conditions, manatees that venture outside of thermal refuges to feed or migrate would be exposed to potentially lethal cold. An absolute trigger point could not be identified in the CW region (using water or air data) or in the CE region (using air data) because the top-ranked models included a winter term. Alternatives for the CW region would be to use the second- or third-ranked model (Table 3) or to qualitatively select a short-term HDD value based on the observed relationships between carcass counts and water (Fig 5, ~80 HDD) or air (Fig 6, ~110 HDD) temperature metrics.

### Assumptions

Our analyses involved several important assumptions that we evaluate here. First, we assumed constant abundance in each region throughout each winter. The categorical winter term (selected in most of our top-ranked models) partly accounts for variation in abundance between winters. Within-winter fluctuations in abundance associated with seasonal distributional shifts are more likely to result in a violation of this assumption. In the CW region, manatees are unlikely to move in or out of the region in winter [43]. Manatee abundance in the CE region is likely to be higher at the start and end of winter and during mild periods due to seasonal migrations [13,25,44], so for a given mortality rate, we would expect more carcasses during warm periods when regional abundance is higher. This is the opposite of what we found, indicating that the cold signal clearly stood out, despite noise in the data created by any variations in abundance within a winter.

Second, we assumed a constant reporting interval (i.e., time from death to reporting) and rate (i.e., proportion of deaths reported) for manatee carcasses in our study area during the winters analyzed. We intentionally selected the two regions where human population is dense all winter and where there is much spatial overlap in manatee distribution and human activity in shallow, inshore estuarine, and riverine habitats [45]. If the carcass of a non-perinatal manatee were present in these areas, its detection was deemed likely. Manatee carcasses are reported to authorities using a well-established wildlife hotline, and no information suggests that reporting to the hotline varied during the period of our study.

We further assumed that the health condition of animals at the start of a winter was the same across all winters. For example, we assumed that a manatee that survived a severe winter (like 2009–2010) was not still compromised at the start of the following winter. If it was, it might succumb to CSS more quickly, and, in turn, the interval between cold event and increased mortality would decrease. There was notable consistency in the short-term temperature effects selected among regions and data set types, suggesting that this assumption was not violated.

Finally, we assumed that the quality of warm-water habitat remained constant both within and across winters in each region. Diminished warm-water habitat could result in an increased number of cold-related carcasses. In the CW region, operations at the two principal power plants used by manatees (TECO Big Bend plant in Tampa Bay and Florida Power and Light Company (FPL) Ft. Myers plant on the Caloosahatchee River) were quite consistent within and across winters, with daily mean discharge temperatures remaining above 20°C nearly continuously throughout all winters of the study. In the CE region, FPL’s Cape Canaveral Power Plant underwent a 3-year modernization process beginning in fall 2010, during which the plant did not operate. An interim warm-water refuge was created specifically for manatees and operated for the subsequent 3 winters, somewhat altering the warm-water habitat in the region [13]. To test whether power plant operation influenced the relationship between cold-related mortality and temperature in the CE region, we replaced the winter effect in the models with a single heating-degree-hour effect (using a base of 20°C, cumulative over the entire winter) based on discharge temperatures at the plant. We retested the models with this new variable, and the top-ranked models remained the same when using water or air data. While this does not rule out the possibility that variation in power plant operation influenced the number of cold-stress carcasses reported, it does not suggest that our results were influenced by changes in the warm-water network.

### Conservation implications

Ongoing and future conservation efforts for the Florida manatee need to ensure that there is sufficient warm-water habitat available to manatees when temperatures reach critical cold thresholds during the winter. Warm-water networks for manatees are likely to contract in the future due to changes in power generation technologies, human development, and population pressures, and possibly climate change [10]. Sea level rise may place portions of coastal power plants, on which many manatees rely for warm water, underwater [31]. Population projections estimate as many as 9 million more people in Florida by 2045 [46], which could further reduce the availability of groundwater and spring flows that provide stable, warm water directly into many winter aggregation sites. Continuing to evaluate the relationship between cold-weather events and cold-related manatee mortality could provide important insight into how manatees respond to changes in their warm-water network.

Our results apply to the central regions of Florida with their warm-water networks, which consist primarily of power plant discharges and passive thermal basins. The northwest and Upper St. Johns River regions of Florida are north of and colder than the CE and CW regions; manatees in those regions use springs as a buffer against the colder temperatures. The remoteness of parts of southwestern Florida could result in fewer carcasses being reported in regions such as the Everglades and greater delays in reports and responses; thus, carcasses are usually more decomposed, and fewer cold stress deaths are determined. There would likely be more time after a cold event before manatees dying from cold in remote areas were reported. The waters in the southeast region of Florida are warmed by the Gulf Stream, so temperatures of ambient water bodies are generally warmer; consequently, manatees in this area are exposed to cold water less than in other regions of Florida and mortality from cold stress is lower.

### Further exploration

Our findings provide a foundation for understanding the temporal relationship between winter temperature patterns and cold-related manatee mortality. Many of our top-ranked models included a winter categorical effect, which suggests that additional factors―such as fluctuations in regional manatee abundance, in manatee distribution, or in quality of warm-water habitat at aggregation sites―may affect the number of cold-related deaths. Further work should be done to refine model input parameters to better elucidate the factors that account for variation in cold-related manatee mortality. Low sample size precluded us from considering age class in the models, but given the greater vulnerability of calves and subadults to cold compared to adults [20,21], such analyses seem worthwhile and are likely to show that different temperature thresholds apply to different age or size classes. The analysis could also be expanded to include more winters and other regions in the Florida manatee’s range. Additionally, future model development should focus on identifying readily accessible temperature data that are available in real-time for use in these models to predict the number of cold-related manatee carcasses that may be expected during cold events.

## Supporting information

S1 Text Temperature patterns and cold-related mortality. (DOCX)

S1 Fig Manatee mortality in Florida, 1989–1990 through 2014–2015, by cause of death. Year is defined as November 1 to October 31, such that each winter is summarized as part of a single time period. During 2009–2010, 248 non-perinatal cold-related deaths were reported when unusually cold temperatures were recorded throughout most of Florida. During 2009–2010 and 2010–2011, 59.8% of non-perinatal mortality for which a cause of death could be determined was due to cold stress.
(TIFF)

S2 Fig Number of cold-related manatee carcasses reported and lagged water temperature effects by week for the central-east (CE) region. Water temperature data are displayed as heating degree days relative to 20°C, both by week and cumulatively throughout the winter.
(TIFF)

S3 Fig Number of cold-related manatee carcasses reported and lagged water temperature effects by week for the central-west (CW) region. Water temperature data are displayed as heating degree days relative to 20°C, both by week and cumulatively throughout the winter.
(TIFF)

S1 Table Pearson’s correlation coefficients for pairings of temperature effects tested in our models. Pairs of variables that had a correlation coefficient > 0.7 or < -0.7 were interpreted as being correlated to a high degree. Only two pairs of effects in the central-west (CW) region when using water data were found to be correlated at that level (in bold): 7-day sum, lagged by 0 days, correlated with 7-day sum, lagged by 7 days; 7-day sum, lagged by 7 days, correlated with 7-day sum, lagged by 14 days.
(DOCX)

S2 Table Model selection for response variables tested to identify which distribution should be used for modeling cold-related mortality in relation to water and air temperature effects. The parameters used for model selection was the same for all regions and temperature types–an interactive effect between winter and a short-term temperature effect (14-day sum lagged by 10 days). Four response-variable distributions were tested: Poisson, negative binomial, zero-inflated Poisson, and zero-inflated negative binomial. For region, CE refers to the central-east region, and CW refers to the central-west region. We used c—hat (sum of squared Pearson chi-square residuals divided by residual degrees of freedom) and chi-square goodness of fit (calculated on the same quantities) to evaluate response variable distributions. Distributions in which c-hat was close to 1.0 (and < 1.2) and the chi-square value was not significant (p > 0.05) were considered to have a good fit to the data.
(DOCX)

S3 Table Models tested for both water and air temperatures. **Number of parameters is given for the Poisson variable distribution**. Model name depicts the terms of the model. The short-term temperature effects were named Sum#_Lag#, and the cumulative temperature effects were named SumCum_Lag (where # indicates the number of days the data were summed or lagged). The full model names include all the terms that were used in the model. + indicates that the effects were additive; * indicates that the effects were interactive.
(DOCX)

S4 Table Number of reported manatees that died from cold stress and cumulative HDD for each region and winter. Cumulative HDD is calculated using daily HDD values from water temperature monitoring locations for each winter from December 1 through March 31.
(DOCX)

S5 Table Suite of 61 models tested for 7-day counts of cold-related manatee carcasses reported in the central-east region based on water temperature. Reports of cold-related carcasses were modeled using a negative binomial generalized linear model, and models were ranked using the AICc value. Temperature variables used in the models are described in Fig 2.
(DOCX)

S6 Table Suite of 61 models tested for 7-day counts of cold-related manatee carcasses reported in the central-east region based on air temperature. Reports of cold-related carcasses were modeled using a negative binomial generalized linear model, and models were ranked using the AICc value. Temperature variables used in the models are described in Fig 2.
(DOCX)

S7 Table Suite of 61 models tested for 7-day counts of cold-related manatee carcasses reported in the central-west region based on water temperature. Reports of cold-related carcasses were modeled using a Poisson generalized linear model, and models were ranked using the AICc value. Temperature variables used in the models are described in Fig 2.
(DOCX)

S8 Table Suite of 61 models tested for 7-day counts of cold-related manatee carcasses reported in the central-west region based on air temperature. Reports of cold-related carcasses were modeled using a Poisson generalized linear model, and models were ranked using the AICc value. Temperature variables used in the models are described in Fig 2.
(DOCX)
