# Supplementary material for: Cold-related Florida manatee mortality in relation to air and water temperatures
Source: PLoS One. 2019 Nov 21;14(11):e0225048. doi: 10.1371/journal.pone.0225048 (PMC6871784; doi:10.1371/journal.pone.0225048)
Supplement: S1 Text — (DOCX) [file pone.0225048.s001.docx]

The overall severity of each winter (December 1–March 31) was quantified using the cumulative HDD values from water temperature. During our 6-year study period, the three winters from 2011–2012 through 2013–2014 were mildest, with cumulative HDD values < 77 in the CE region and < 141 in the CW region. Winter 2008–2009 was moderately cold, with cumulative HDD values that were 2.2 and 2.1 times the average cumulative HDD of the three mild winters in the CE and CW regions, respectively. Winters 2009–2010 and 2010–2011 were severely cold, with cumulative HDD values that were as much as 4.6 and 3.8 times the average HDD of the mild winters in the CE and CW regions, respectively (S4 Table, S2 Fig). The colder the winter, the more cold-related carcasses reported, with R^2^ values of 0.898 and 0.910 for the CE and CW regions, respectively, using a linear relationship between carcass count and cumulative HDD. During four mild or moderate winters, no more than six carcasses attributable to cold were reported in a 7-day window in the CE region and no more than two carcasses in the CW region (S2 Fig). In contrast, in mid-January 2010 (the coldest period of these 6 winters), the largest number of carcasses reported in a 7-day period was 42 in the CE region and 10 in the CW region. Cold-related manatee mortality spiked again when temperatures dropped in March and remained elevated into April 2010 (S2 Fig) [5]. During winter 2010–2011, a series of strong cold fronts arrived during December, and temperatures remained below normal through January. Large numbers of cold-related carcasses were reported during this period (3–10 per 7-day period in the CE region and 1-9 per 7-day period in the CW region).
