# Supplementary figures and images for: Cold-related Florida manatee mortality in relation to air and water temperatures
Source: PLoS One. 2019 Nov 21;14(11):e0225048. doi: 10.1371/journal.pone.0225048 (PMC6871784; doi:10.1371/journal.pone.0225048)

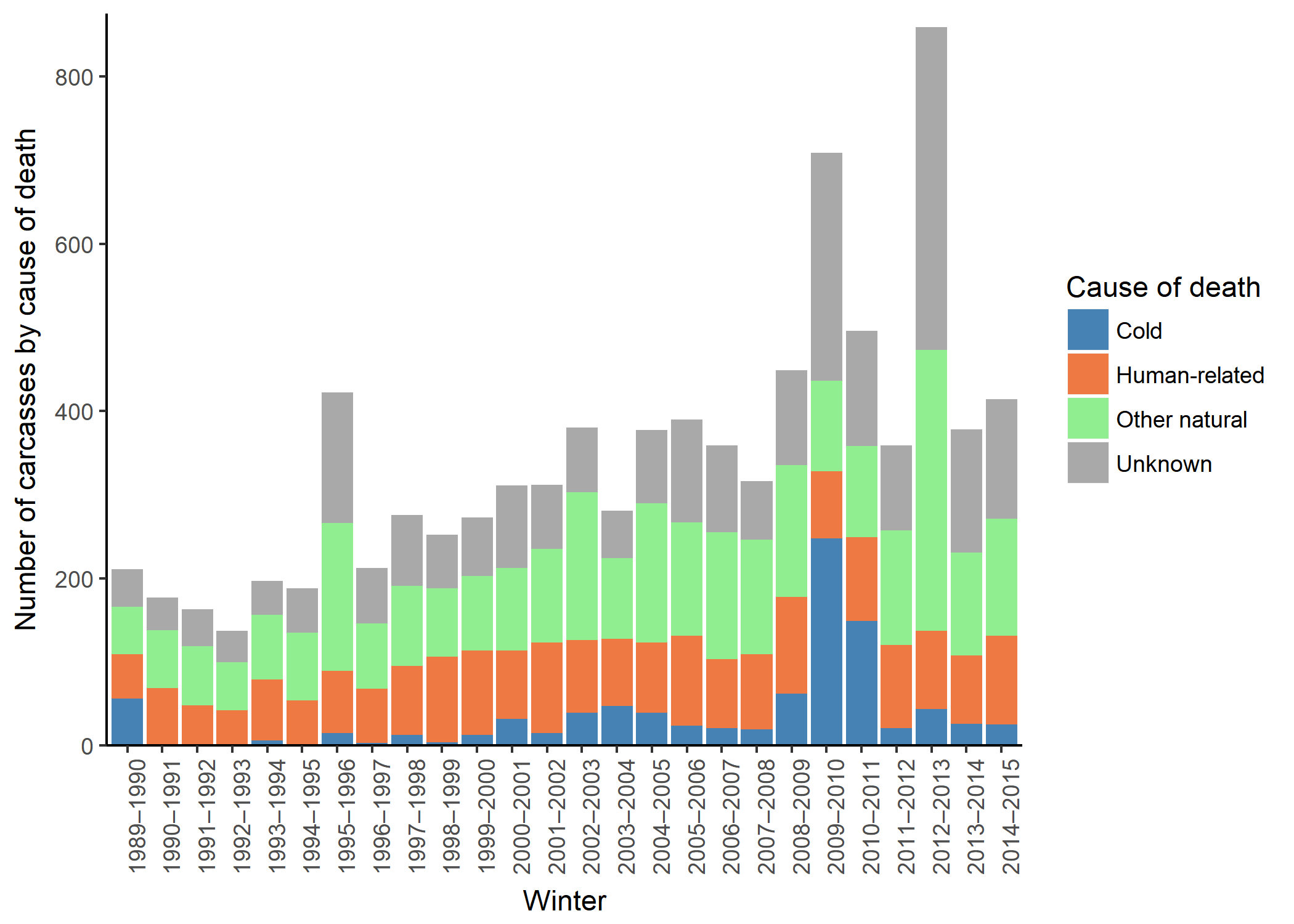

Supplement: S1 Fig — Year is defined as November 1 to October 31, such that each winter is summarized as part of a single time period. During 2009–2010, 248 non-perinatal cold-related deaths were reported when unusually cold temperatures were recorded throughout most of Florida. During 2009–2010 and 2010–2011, 59.8% of non-perinatal mortality for which a cause of death could be determined was due to cold stress. (TIFF) [file pone.0225048.s002.tiff]

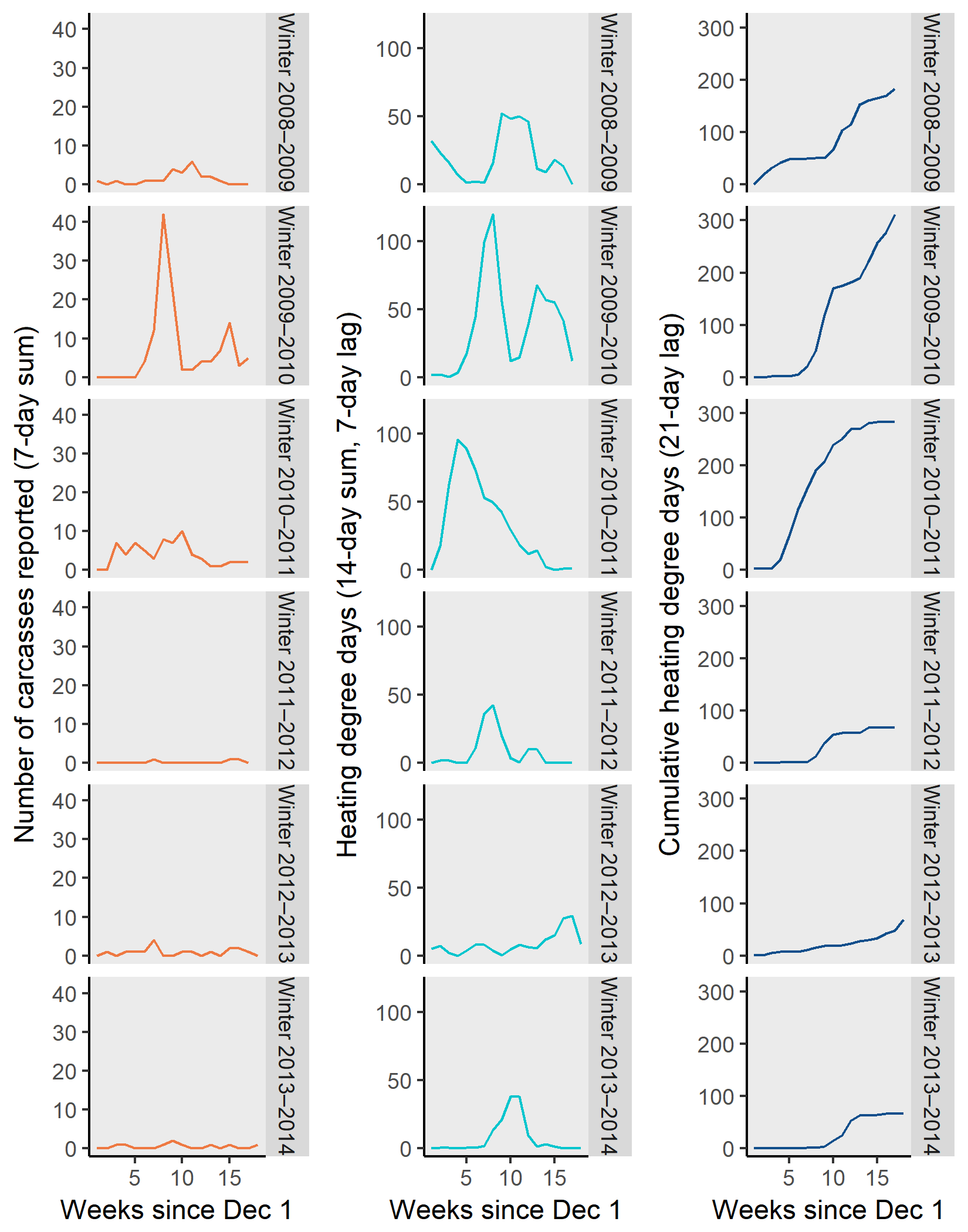

Supplement: S2 Fig — Water temperature data are displayed as heating degree days relative to 20°C, both by week and cumulatively throughout the winter. (TIFF) [file pone.0225048.s003.tiff]

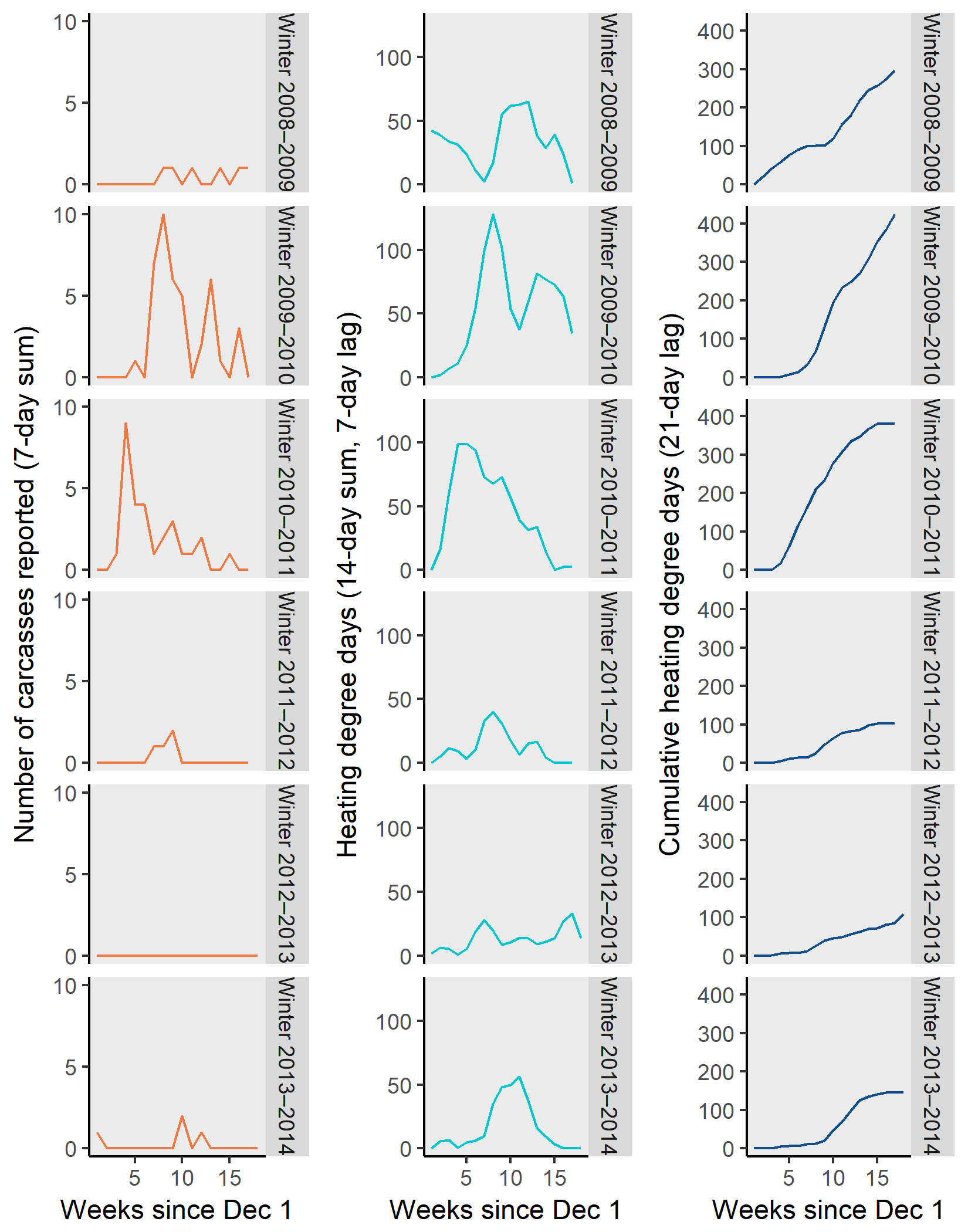

Supplement: S3 Fig — Water temperature data are displayed as heating degree days relative to 20°C, both by week and cumulatively throughout the winter. (TIFF) [file pone.0225048.s004.tiff]
