# Supplementary material for: Cold-related Florida manatee mortality in relation to air and water temperatures
Source: PLoS One. 2019 Nov 21;14(11):e0225048. doi: 10.1371/journal.pone.0225048 (PMC6871784; doi:10.1371/journal.pone.0225048)
Supplement: S1 Table — Pairs of variables that had a correlation coefficient > 0.7 or < -0.7 were interpreted as being correlated to a high degree. Only two pairs of effects in the central-west (CW) region when using water data were found to be correlated at that level (in bold): 7-day sum, lagged by 0 days, correlated with 7-day sum, lagged by 7 days; 7-day sum, lagged by 7 days, correlated with 7-day sum, lagged by 14 days. (DOCX) [file pone.0225048.s005.docx]

|  |  | | Pearson’s correlation coefficient | | | |
| --- | --- | --- | --- | --- | --- | --- |
| Effect 1 | | **Effect 2** | **CE**  **Air** | **CE Water** | **CW**  **Air** | **CW Water** |
| HDD_Sum7_Lag0 | | HDD_Sum7_Lag7 | 0.534 | 0.632 | 0.562 | **0.776** |
| HDD_Sum7_Lag0 | | HDD_Sum7_Lag14 | 0.324 | 0.371 | 0.349 | 0.528 |
| HDD_Sum7_Lag0 | | HDD_Sum14_Lag7 | 0.490 | 0.555 | 0.515 | 0.691 |
| HDD_Sum7_Lag0 | | HDD_SumCum_Lag21 | -0.030 | -0.063 | -0.119 | -0.045 |
| HDD_Sum7_Lag7 | | HDD_Sum7_Lag14 | 0.530 | 0.632 | 0.561 | **0.778** |
| HDD_Sum7_Lag7 | | HDD_SumCum_Lag21 | 0.095 | 0.055 | 0.025 | 0.117 |
| HDD_Sum7_Lag14 | | HDD_SumCum_Lag21 | 0.242 | 0.207 | 0.199 | 0.308 |
| HDD_Sum14_Lag7 | | HDD_SumCum_Lag21 | 0.193 | 0.145 | 0.127 | 0.223 |
| HDD_Sum14_Lag10 | | HDD_SumCum_Lag24 | 0.237 | 0.185 | 0.186 | 0.276 |
