# Supplementary material for: Cold-related Florida manatee mortality in relation to air and water temperatures
Source: PLoS One. 2019 Nov 21;14(11):e0225048. doi: 10.1371/journal.pone.0225048 (PMC6871784; doi:10.1371/journal.pone.0225048)
Supplement: S2 Table — The parameters used for model selection was the same for all regions and temperature types–an interactive effect between winter and a short-term temperature effect (14-day sum lagged by 10 days). Four response-variable distributions were tested: Poisson, negative binomial, zero-inflated Poisson, and zero-inflated negative binomial. For region, CE refers to the central-east region, and CW refers to the central-west region. We used c—hat (sum of squared Pearson chi-square residuals divided by residual degrees of freedom) and chi-square goodness of fit (calculated on the same quantities) to evaluate response variable distributions. Distributions in which c-hat was close to 1.0 (and < 1.2) and the chi-square value was not significant (p > 0.05) were considered to have a good fit to the data. (DOCX) [file pone.0225048.s006.docx]

| Region | Data Type | Model | No. of parameters | ΔAICc | Weight | c-hat | Goodness of Fit |
| --- | --- | --- | --- | --- | --- | --- | --- |
| CE | Water | Negative binomial | 13 | 0.000 | 0.546 | 1.015 | 0.440 |
| CE | Water | Poisson | 12 | 1.651 | 0.239 | 1.272 | 0.040 |
| CE | Water | Zero-inflated negative binomial | 14 | 2.675 | 0.144 | 1.038 | 0.382 |
| CE | Water | Zero-inflated Poisson | 13 | 4.088 | 0.071 | 1.196 | 0.098 |
| CE | Air | Negative binomial | 13 | 0.000 | 0.788 | 0.924 | 0.684 |
| CE | Air | Zero-inflated negative binomial | 14 | 2.675 | 0.208 | 0.944 | 0.629 |
| CE | Air | Poisson | 12 | 10.808 | 0.004 | 1.400 | 0.007 |
| CE | Air | Zero-inflated Poisson | 13 | 13.425 | 0.000 | 1.416 | 0.006 |
| CW | Water | Poisson | 12 | 0.000 | 0.479 | 0.833 | 0.876 |
| CW | Water | Negative binomial | 13 | 0.875 | 0.310 | 0.702 | 0.987 |
| CW | Water | Zero-inflated Poisson | 13 | 2.616 | 0.130 | 0.841 | 0.861 |
| CW | Water | Zero-inflated negative binomial | 14 | 3.550 | 0.081 | 0.718 | 0.980 |
| CW | Air | Poisson | 12 | 0.000 | 0.621 | 0.940 | 0.642 |
| CW | Air | Zero-inflated Poisson | 13 | 2.616 | 0.168 | 0.948 | 0.619 |
| CW | Air | Negative binomial | 13 | 2.617 | 0.168 | 0.940 | 0.642 |
| CW | Air | Zero-inflated negative binomial | 14 | 5.290 | 0.044 | 0.960 | 0.588 |
