# Supplementary material for: Cold-related Florida manatee mortality in relation to air and water temperatures
Source: PLoS One. 2019 Nov 21;14(11):e0225048. doi: 10.1371/journal.pone.0225048 (PMC6871784; doi:10.1371/journal.pone.0225048)
Supplement: S3 Table — Number of parameters is given for the Poisson variable distribution. Model name depicts the terms of the model. The short-term temperature effects were named Sum#_Lag#, and the cumulative temperature effects were named SumCum_Lag (where # indicates the number of days the data were summed or lagged). The full model names include all the terms that were used in the model. + indicates that the effects were additive; * indicates that the effects were interactive. (DOCX) [file pone.0225048.s007.docx]

| Model | No. of parameters |
| --- | --- |
| NULL | 1 |
| Winter | 6 |
| Sum7_Lag0 | 2 |
| Sum7_Lag7 | 2 |
| Sum14_Lag7 | 2 |
| Sum14_Lag10 | 2 |
| Sum7_Lag14 | 2 |
| SumCum_Lag21 | 2 |
| SumCum_Lag24 | 2 |
| Winter + Sum7_Lag0 | 7 |
| Winter + Sum7_Lag7 | 7 |
| Winter + Sum14_Lag7 | 7 |
| Winter + Sum14_Lag10 | 7 |
| Winter + Sum7_Lag14 | 7 |
| Winter + SumCum_Lag21 | 7 |
| Winter + SumCum_Lag24 | 7 |
| Winter * Sum7_Lag0 | 12 |
| Winter * Sum7_Lag7 | 12 |
| Winter * Sum14_Lag7 | 12 |
| Winter * Sum14_Lag10 | 12 |
| Winter * Sum7_Lag14 | 12 |
| Winter * SumCum_Lag21 | 12 |
| Winter * SumCum_Lag24 | 12 |
| Sum7_Lag0 + Sum7_Lag7 | 3 |
| Sum7_Lag0 + Sum7_Lag7 + Sum7_Lag14 | 4 |
| Sum7_Lag0 + Sum7_Lag7 + Sum7_Lag14 + SumCum_Lag21 | 5 |
| Sum7_Lag0 + Sum14_Lag7 | 3 |
| Sum7_Lag0 + Sum14_Lag7 + SumCum_Lag21 | 4 |
| Sum7_Lag7 + Sum7_Lag14 | 3 |
| Sum7_Lag7 + Sum7_Lag14 + SumCum_Lag21 | 4 |
| Sum7_Lag7 + SumCum_Lag21 | 3 |
| Sum14_Lag7 + SumCum_Lag21 | 3 |
| Sum14_Lag10 + SumCum_Lag24 | 3 |
| Sum7_Lag14 + SumCum_Lag21 | 3 |
| Sum7_Lag0 * Sum7_Lag7 | 4 |
| Sum7_Lag0 * Sum14_Lag7 | 4 |
| Sum7_Lag0 * Sum7_Lag14 | 4 |
| Sum7_Lag7 * Sum7_Lag14 | 4 |
| Sum7_Lag7 * SumCum_Lag21 | 4 |
| Sum14_Lag7 * SumCum_Lag21 | 4 |
| Sum14_Lag10 * SumCum_Lag24 | 4 |
| Sum7_Lag14 * SumCum_Lag21 | 4 |
| Winter + Sum7_Lag0 + Sum7_Lag7 | 8 |
| Winter + Sum7_Lag0 + Sum7_Lag7 + Sum7_Lag14 | 9 |
| Winter + Sum7_Lag0 + Sum7_Lag7 + Sum7_Lag14 + SumCum_Lag21 | 10 |
| Winter + Sum7_Lag0 + Sum14_Lag7 | 8 |
| Winter + Sum7_Lag0 + Sum14_Lag7 + SumCum_Lag21 | 9 |
| Winter + Sum7_Lag7 + Sum7_Lag14 | 8 |
| Winter + Sum7_Lag7 + Sum7_Lag14 + SumCum_Lag21 | 9 |
| Winter + Sum7_Lag7 + SumCum_Lag21 | 8 |
| Winter + Sum14_Lag7 + SumCum_Lag21 | 8 |
| Winter + Sum14_Lag10 + SumCum_Lag24 | 8 |
| Winter + Sum7_Lag14 + SumCum_Lag21 | 8 |
| Winter + Sum7_Lag0 * Sum7_Lag7 | 9 |
| Winter + Sum7_Lag0 * Sum14_Lag7 | 9 |
| Winter + Sum7_Lag0 * Sum7_Lag14 | 9 |
| Winter + Sum7_Lag7 * Sum7_Lag14 | 9 |
| Winter + Sum7_Lag7 * SumCum_Lag21 | 9 |
| Winter + Sum14_Lag7 * SumCum_Lag21 | 9 |
| Winter + Sum14_Lag10 * SumCum_Lag24 | 9 |
| Winter + Sum7_Lag14 * SumCum_Lag21 | 9 |
