# Supplementary material for: Cold-related Florida manatee mortality in relation to air and water temperatures
Source: PLoS One. 2019 Nov 21;14(11):e0225048. doi: 10.1371/journal.pone.0225048 (PMC6871784; doi:10.1371/journal.pone.0225048)
Supplement: S4 Table — Cumulative HDD is calculated using daily HDD values from water temperature monitoring locations for each winter from December 1 through March 31. (DOCX) [file pone.0225048.s008.docx]

| Winter | Central-east (CE) region | | Central-west (CW) region | |
| --- | --- | --- | --- | --- |
|  | **No. of carcasses** | **Cumulative HDD** | **No. of carcasses** | **Cumulative HDD** |
| 2008–2009 | 23 | 151.65 | 6 | 255.46 |
| 2009–2010 | 121 | 322.13 | 41 | 462.94 |
| 2010–2011 | 66 | 281.93 | 29 | 382.12 |
| 2011–2012 | 3 | 67.60 | 4 | 101.58 |
| 2012–2013 | 16 | 76.77 | 0 | 124.12 |
| 2013–2014 | 9 | 65.33 | 4 | 140.54 |
