# Supplementary material for: Cold-related Florida manatee mortality in relation to air and water temperatures
Source: PLoS One. 2019 Nov 21;14(11):e0225048. doi: 10.1371/journal.pone.0225048 (PMC6871784; doi:10.1371/journal.pone.0225048)
Supplement: S5 Table — Reports of cold-related carcasses were modeled using a negative binomial generalized linear model, and models were ranked using the AICc value. Temperature variables used in the models are described in Fig 2. (DOCX) [file pone.0225048.s009.docx]

| Model | No. of parameters | AICc | ΔAICc | Weight |
| --- | --- | --- | --- | --- |
| Sum14_Lag7 + SumCum_Lag21 | 4 | 303.008 | 0.000 | 0.265 |
| Winter + Sum14_Lag7 + SumCum_Lag21 | 9 | 303.818 | 0.809 | 0.177 |
| Sum7_Lag7 + Sum7_Lag14 + SumCum_Lag21 | 5 | 305.070 | 2.062 | 0.094 |
| Sum7_Lag0 + Sum14_Lag7 + SumCum_Lag21 | 5 | 305.191 | 2.183 | 0.090 |
| Sum14_Lag7 * SumCum_Lag21 | 5 | 305.212 | 2.203 | 0.088 |
| Winter + Sum7_Lag7 + Sum7_Lag14 + SumCum_Lag21 | 10 | 306.136 | 3.127 | 0.055 |
| Winter + Sum7_Lag0 + Sum14_Lag7 + SumCum_Lag21 | 10 | 306.143 | 3.135 | 0.055 |
| Winter + Sum14_Lag7 * SumCum_Lag21 | 10 | 306.170 | 3.162 | 0.054 |
| Winter + Sum14_Lag10 + SumCum_Lag24 | 9 | 307.157 | 4.148 | 0.033 |
| Sum7_Lag0 + Sum7_Lag7 + Sum7_Lag14 + SumCum_Lag21 | 6 | 307.227 | 4.218 | 0.032 |
| Sum14_Lag10 + SumCum_Lag24 | 4 | 308.443 | 5.435 | 0.017 |
| Winter + Sum7_Lag0 + Sum7_Lag7 + Sum7_Lag14 + SumCum_Lag21 | 11 | 308.579 | 5.571 | 0.016 |
| Winter + Sum14_Lag10 * SumCum_Lag24 | 10 | 309.183 | 6.175 | 0.012 |
| Sum14_Lag10 * SumCum_Lag24 | 5 | 310.346 | 7.337 | 0.007 |
| Winter + Sum14_Lag10 | 8 | 313.253 | 10.245 | 0.001 |
| Winter + Sum7_Lag0 * Sum14_Lag7 | 10 | 315.119 | 12.111 | 0.000 |
| Winter + Sum7_Lag0 + Sum14_Lag7 | 9 | 315.277 | 12.268 | 0.000 |
| Winter + Sum7_Lag7 * Sum7_Lag14 | 10 | 316.337 | 13.328 | 0.000 |
| Winter * Sum14_Lag10 | 13 | 316.969 | 13.961 | 0.000 |
| Winter + Sum14_Lag7 | 8 | 317.315 | 14.307 | 0.000 |
| Winter + Sum7_Lag0 + Sum7_Lag7 + Sum7_Lag14 | 10 | 317.704 | 14.695 | 0.000 |
| Winter + Sum7_Lag7 + Sum7_Lag14 | 9 | 318.976 | 15.967 | 0.000 |
| Winter * Sum14_Lag7 | 13 | 323.142 | 20.134 | 0.000 |
| Winter + Sum7_Lag14 * SumCum_Lag21 | 10 | 324.083 | 21.075 | 0.000 |
| Winter + Sum7_Lag14 + SumCum_Lag21 | 9 | 324.201 | 21.193 | 0.000 |
| Winter + Sum7_Lag14 | 8 | 326.783 | 23.775 | 0.000 |
| Sum14_Lag10 | 3 | 329.145 | 26.137 | 0.000 |
| Sum7_Lag14 * SumCum_Lag21 | 5 | 329.537 | 26.528 | 0.000 |
| Winter + Sum7_Lag7 + SumCum_Lag21 | 9 | 329.694 | 26.686 | 0.000 |
| Sum7_Lag14 + SumCum_Lag21 | 4 | 329.808 | 26.800 | 0.000 |
| Sum7_Lag7 * Sum7_Lag14 | 5 | 330.639 | 27.631 | 0.000 |
| Winter + Sum7_Lag0 * Sum7_Lag14 | 10 | 330.723 | 27.715 | 0.000 |
| Winter + Sum7_Lag0 * Sum7_Lag7 | 10 | 331.858 | 28.849 | 0.000 |
| Winter + Sum7_Lag7 * SumCum_Lag21 | 10 | 331.941 | 28.933 | 0.000 |
| Sum14_Lag7 | 3 | 333.551 | 30.542 | 0.000 |
| Winter + Sum7_Lag0 + Sum7_Lag7 | 9 | 334.118 | 31.110 | 0.000 |
| Sum7_Lag0 * Sum14_Lag7 | 5 | 334.131 | 31.122 | 0.000 |
| Winter * Sum7_Lag14 | 13 | 334.631 | 31.623 | 0.000 |
| Sum7_Lag0 + Sum14_Lag7 | 4 | 334.801 | 31.792 | 0.000 |
| Sum7_Lag7 + Sum7_Lag14 | 4 | 334.960 | 31.952 | 0.000 |
| Winter + Sum7_Lag7 | 8 | 335.289 | 32.280 | 0.000 |
| Sum7_Lag0 + Sum7_Lag7 + Sum7_Lag14 | 5 | 336.644 | 33.636 | 0.000 |
| Sum7_Lag7 + SumCum_Lag21 | 4 | 336.991 | 33.983 | 0.000 |
| Sum7_Lag7 * SumCum_Lag21 | 5 | 338.937 | 35.929 | 0.000 |
| Winter * Sum7_Lag7 | 13 | 345.911 | 42.903 | 0.000 |
| Sum7_Lag14 | 3 | 348.434 | 45.426 | 0.000 |
| Sum7_Lag0 * Sum7_Lag14 | 5 | 349.906 | 46.898 | 0.000 |
| Winter | 7 | 355.012 | 52.003 | 0.000 |
| Winter + Sum7_Lag0 | 8 | 357.037 | 54.028 | 0.000 |
| Winter + SumCum_Lag24 | 8 | 357.217 | 54.209 | 0.000 |
| Winter + SumCum_Lag21 | 8 | 357.360 | 54.352 | 0.000 |
| Sum7_Lag0 * Sum7_Lag7 | 5 | 362.553 | 59.545 | 0.000 |
| Sum7_Lag7 | 3 | 363.495 | 60.487 | 0.000 |
| Sum7_Lag0 + Sum7_Lag7 | 4 | 365.475 | 62.467 | 0.000 |
| Winter * Sum7_Lag0 | 13 | 365.542 | 62.534 | 0.000 |
| Winter * SumCum_Lag24 | 13 | 368.842 | 65.834 | 0.000 |
| Winter * SumCum_Lag21 | 13 | 369.186 | 66.178 | 0.000 |
| SumCum_Lag21 | 3 | 396.434 | 93.425 | 0.000 |
| SumCum_Lag24 | 3 | 399.280 | 96.271 | 0.000 |
| Sum7_Lag0 | 3 | 400.207 | 97.199 | 0.000 |
| NULL | 2 | 403.599 | 100.591 | 0.000 |
