# Supplementary material for: Cold-related Florida manatee mortality in relation to air and water temperatures
Source: PLoS One. 2019 Nov 21;14(11):e0225048. doi: 10.1371/journal.pone.0225048 (PMC6871784; doi:10.1371/journal.pone.0225048)
Supplement: S6 Table — Reports of cold-related carcasses were modeled using a negative binomial generalized linear model, and models were ranked using the AICc value. Temperature variables used in the models are described in Fig 2. (DOCX) [file pone.0225048.s010.docx]

| Model | No. of  parameters | AICc | ΔAICc | Weight |
| --- | --- | --- | --- | --- |
| Winter + Sum14_Lag10 + SumCum_Lag24 | 9 | 305.018 | 0.000 | 0.419 |
| Winter + Sum14_Lag10 * SumCum_Lag24 | 10 | 306.484 | 1.466 | 0.201 |
| Sum14_Lag10 * SumCum_Lag24 | 5 | 307.226 | 2.207 | 0.139 |
| Winter + Sum14_Lag10 | 8 | 307.535 | 2.517 | 0.119 |
| Sum14_Lag10 + SumCum_Lag24 | 4 | 307.600 | 2.582 | 0.115 |
| Winter * Sum14_Lag10 | 13 | 315.447 | 10.429 | 0.002 |
| Winter + Sum7_Lag7 + Sum7_Lag14 + SumCum_Lag21 | 10 | 318.552 | 13.533 | 0.000 |
| Sum7_Lag7 + Sum7_Lag14 + SumCum_Lag21 | 5 | 319.192 | 14.174 | 0.000 |
| Winter + Sum14_Lag7 + SumCum_Lag21 | 9 | 319.458 | 14.440 | 0.000 |
| Sum14_Lag7 + SumCum_Lag21 | 4 | 319.642 | 14.624 | 0.000 |
| Winter + Sum7_Lag0 + Sum14_Lag7 + SumCum_Lag21 | 10 | 320.060 | 15.042 | 0.000 |
| Winter + Sum7_Lag0 * Sum14_Lag7 | 10 | 320.117 | 15.099 | 0.000 |
| Winter + Sum7_Lag0 + Sum7_Lag7 + Sum7_Lag14 + SumCum_Lag21 | 11 | 320.163 | 15.145 | 0.000 |
| Sum14_Lag7 * SumCum_Lag21 | 5 | 320.490 | 15.472 | 0.000 |
| Sum14_Lag10 | 3 | 320.789 | 15.771 | 0.000 |
| Winter + Sum14_Lag7 * SumCum_Lag21 | 10 | 321.174 | 16.156 | 0.000 |
| Sum7_Lag0 + Sum7_Lag7 + Sum7_Lag14 + SumCum_Lag21 | 6 | 321.368 | 16.350 | 0.000 |
| Winter + Sum7_Lag0 + Sum14_Lag7 | 9 | 321.748 | 16.730 | 0.000 |
| Sum7_Lag0 + Sum14_Lag7 + SumCum_Lag21 | 5 | 321.833 | 16.815 | 0.000 |
| Winter + Sum7_Lag7 * Sum7_Lag14 | 10 | 321.972 | 16.953 | 0.000 |
| Winter + Sum7_Lag0 + Sum7_Lag7 + Sum7_Lag14 | 10 | 322.013 | 16.995 | 0.000 |
| Winter + Sum7_Lag14 * SumCum_Lag21 | 10 | 322.221 | 17.203 | 0.000 |
| Winter + Sum7_Lag7 + Sum7_Lag14 | 9 | 322.517 | 17.499 | 0.000 |
| Winter + Sum7_Lag14 + SumCum_Lag21 | 9 | 323.048 | 18.030 | 0.000 |
| Winter + Sum14_Lag7 | 8 | 323.784 | 18.765 | 0.000 |
| Winter + Sum7_Lag14 | 8 | 324.493 | 19.474 | 0.000 |
| Sum7_Lag14 * SumCum_Lag21 | 5 | 326.948 | 21.930 | 0.000 |
| Winter + Sum7_Lag0 * Sum7_Lag14 | 10 | 328.326 | 23.307 | 0.000 |
| Sum7_Lag14 + SumCum_Lag21 | 4 | 329.078 | 24.060 | 0.000 |
| Winter * Sum14_Lag7 | 13 | 333.657 | 28.638 | 0.000 |
| Winter * Sum7_Lag14 | 13 | 334.169 | 29.150 | 0.000 |
| Sum7_Lag7 * Sum7_Lag14 | 5 | 334.848 | 29.830 | 0.000 |
| Sum7_Lag7 + Sum7_Lag14 | 4 | 337.253 | 32.235 | 0.000 |
| Sum7_Lag0 * Sum14_Lag7 | 5 | 337.589 | 32.571 | 0.000 |
| Sum14_Lag7 | 3 | 338.184 | 33.166 | 0.000 |
| Sum7_Lag0 + Sum7_Lag7 + Sum7_Lag14 | 5 | 339.358 | 34.340 | 0.000 |
| Sum7_Lag0 + Sum14_Lag7 | 4 | 339.871 | 34.853 | 0.000 |
| Winter + Sum7_Lag0 * Sum7_Lag7 | 10 | 342.299 | 37.280 | 0.000 |
| Sum7_Lag14 | 3 | 344.328 | 39.310 | 0.000 |
| Winter + Sum7_Lag0 + Sum7_Lag7 | 9 | 344.592 | 39.573 | 0.000 |
| Winter + Sum7_Lag7 | 8 | 345.290 | 40.271 | 0.000 |
| Winter + Sum7_Lag7 + SumCum_Lag21 | 9 | 345.801 | 40.783 | 0.000 |
| Sum7_Lag0 * Sum7_Lag14 | 5 | 347.325 | 42.307 | 0.000 |
| Winter + Sum7_Lag7 * SumCum_Lag21 | 10 | 348.177 | 43.159 | 0.000 |
| Winter | 7 | 355.012 | 49.994 | 0.000 |
| Winter * Sum7_Lag7 | 13 | 355.580 | 50.562 | 0.000 |
| Winter + Sum7_Lag0 | 8 | 357.219 | 52.201 | 0.000 |
| Winter + SumCum_Lag21 | 8 | 357.285 | 52.267 | 0.000 |
| Winter + SumCum_Lag24 | 8 | 357.355 | 52.337 | 0.000 |
| Sum7_Lag7 + SumCum_Lag21 | 4 | 358.993 | 53.974 | 0.000 |
| Sum7_Lag7 * SumCum_Lag21 | 5 | 360.613 | 55.594 | 0.000 |
| Winter * Sum7_Lag0 | 13 | 365.260 | 60.242 | 0.000 |
| Winter * SumCum_Lag21 | 13 | 368.509 | 63.491 | 0.000 |
| Winter * SumCum_Lag24 | 13 | 368.803 | 63.785 | 0.000 |
| Sum7_Lag0 * Sum7_Lag7 | 5 | 370.414 | 65.396 | 0.000 |
| Sum7_Lag7 | 3 | 373.376 | 68.358 | 0.000 |
| Sum7_Lag0 + Sum7_Lag7 | 4 | 375.528 | 70.510 | 0.000 |
| SumCum_Lag21 | 3 | 395.419 | 90.400 | 0.000 |
| SumCum_Lag24 | 3 | 398.981 | 93.963 | 0.000 |
| Sum7_Lag0 | 3 | 401.362 | 96.344 | 0.000 |
| NULL | 2 | 403.599 | 98.581 | 0.000 |
