# Supplementary material for: Cold-related Florida manatee mortality in relation to air and water temperatures
Source: PLoS One. 2019 Nov 21;14(11):e0225048. doi: 10.1371/journal.pone.0225048 (PMC6871784; doi:10.1371/journal.pone.0225048)
Supplement: S7 Table — Reports of cold-related carcasses were modeled using a Poisson generalized linear model, and models were ranked using the AICc value. Temperature variables used in the models are described in Fig 2. (DOCX) [file pone.0225048.s011.docx]

| Model | No. of parameters | AICc | ΔAICc | Weight |
| --- | --- | --- | --- | --- |
| Winter + Sum14_Lag7 | 7 | 171.084 | 0.000 | 0.145 |
| Sum14_Lag7 * SumCum_Lag21 | 4 | 171.888 | 0.804 | 0.097 |
| Sum14_Lag7 + SumCum_Lag21 | 3 | 172.091 | 1.007 | 0.088 |
| Sum7_Lag0 + Sum14_Lag7 + SumCum_Lag21 | 4 | 172.573 | 1.489 | 0.069 |
| Sum7_Lag7 * Sum7_Lag14 | 4 | 172.676 | 1.592 | 0.065 |
| Sum14_Lag7 | 2 | 172.916 | 1.832 | 0.058 |
| Winter + Sum14_Lag7 + SumCum_Lag21 | 8 | 173.133 | 2.049 | 0.052 |
| Sum7_Lag7 + Sum7_Lag14 + SumCum_Lag21 | 4 | 173.203 | 2.119 | 0.050 |
| Winter + Sum7_Lag7 + Sum7_Lag14 | 8 | 173.241 | 2.157 | 0.049 |
| Winter + Sum7_Lag0 + Sum14_Lag7 | 8 | 173.422 | 2.338 | 0.045 |
| Winter + Sum7_Lag7 * Sum7_Lag14 | 9 | 173.756 | 2.672 | 0.038 |
| Winter * Sum14_Lag7 | 12 | 173.920 | 2.836 | 0.035 |
| Winter + Sum14_Lag7 * SumCum_Lag21 | 9 | 174.109 | 3.026 | 0.032 |
| Sum7_Lag0 + Sum14_Lag7 | 3 | 174.425 | 3.341 | 0.027 |
| Sum7_Lag0 + Sum7_Lag7 + Sum7_Lag14 + SumCum_Lag21 | 5 | 174.608 | 3.525 | 0.025 |
| Sum7_Lag7 + Sum7_Lag14 | 3 | 174.803 | 3.719 | 0.022 |
| Winter + Sum7_Lag7 + Sum7_Lag14 + SumCum_Lag21 | 9 | 175.040 | 3.956 | 0.020 |
| Winter + Sum7_Lag0 + Sum14_Lag7 + SumCum_Lag21 | 9 | 175.330 | 4.246 | 0.017 |
| Winter + Sum7_Lag0 + Sum7_Lag7 + Sum7_Lag14 | 9 | 175.600 | 4.516 | 0.015 |
| Sum7_Lag0 * Sum14_Lag7 | 4 | 175.715 | 4.631 | 0.014 |
| Winter + Sum7_Lag0 * Sum14_Lag7 | 9 | 175.744 | 4.660 | 0.014 |
| Sum7_Lag0 + Sum7_Lag7 + Sum7_Lag14 | 4 | 176.587 | 5.503 | 0.009 |
| Winter + Sum7_Lag0 + Sum7_Lag7 + Sum7_Lag14 + SumCum_Lag21 | 10 | 177.476 | 6.393 | 0.006 |
| Winter + Sum14_Lag10 | 7 | 181.775 | 10.691 | 0.001 |
| Winter + Sum7_Lag0 + Sum7_Lag7 | 6 | 181.813 | 10.729 | 0.001 |
| Winter * Sum14_Lag10 | 12 | 182.383 | 11.299 | 0.001 |
| Winter + Sum7_Lag7 | 7 | 182.388 | 11.304 | 0.001 |
| Winter + Sum14_Lag10 * SumCum_Lag24 | 9 | 182.867 | 11.783 | 0.000 |
| Winter + Sum14_Lag10 + SumCum_Lag24 | 8 | 183.104 | 12.020 | 0.000 |
| Winter + Sum7_Lag7 + SumCum_Lag21 | 8 | 183.275 | 12.191 | 0.000 |
| Winter + Sum7_Lag0 * Sum7_Lag7 | 9 | 183.648 | 12.564 | 0.000 |
| Sum7_Lag7 * SumCum_Lag21 | 4 | 183.683 | 12.599 | 0.000 |
| Sum7_Lag7 + SumCum_Lag21 | 3 | 184.102 | 13.018 | 0.000 |
| Winter + Sum7_Lag7 * SumCum_Lag21 | 9 | 184.555 | 13.472 | 0.000 |
| Winter + Sum7_Lag0 * Sum7_Lag14 | 9 | 185.663 | 14.579 | 0.000 |
| Sum7_Lag0 * Sum7_Lag14 | 4 | 186.049 | 14.965 | 0.000 |
| Sum14_Lag10 | 2 | 186.148 | 15.064 | 0.000 |
| Sum14_Lag10 * SumCum_Lag24 | 4 | 186.394 | 15.310 | 0.000 |
| Winter + Sum7_Lag14 * SumCum_Lag21 | 9 | 186.686 | 15.602 | 0.000 |
| Winter + Sum7_Lag14 | 7 | 187.872 | 16.788 | 0.000 |
| Sum14_Lag10 + SumCum_Lag24 | 3 | 188.080 | 16.996 | 0.000 |
| Winter + Sum7_Lag14 + SumCum_Lag21 | 8 | 188.108 | 17.024 | 0.000 |
| Winter * Sum7_Lag7 | 12 | 188.627 | 17.544 | 0.000 |
| Sum7_Lag7 | 2 | 189.680 | 18.597 | 0.000 |
| Winter * Sum7_Lag14 | 12 | 190.189 | 19.105 | 0.000 |
| Sum7_Lag0 * Sum7_Lag7 | 4 | 190.546 | 19.462 | 0.000 |
| Sum7_Lag0 + Sum7_Lag7 | 3 | 190.737 | 19.654 | 0.000 |
| Sum7_Lag14 * SumCum_Lag21 | 4 | 195.983 | 24.899 | 0.000 |
| Sum7_Lag14 | 2 | 197.507 | 26.424 | 0.000 |
| Sum7_Lag14 + SumCum_Lag21 | 3 | 199.567 | 28.483 | 0.000 |
| Winter + Sum7_Lag0 | 7 | 232.538 | 61.454 | 0.000 |
| Winter * Sum7_Lag0 | 12 | 242.103 | 71.019 | 0.000 |
| Winter + SumCum_Lag24 | 7 | 247.114 | 76.030 | 0.000 |
| Winter + SumCum_Lag21 | 7 | 248.417 | 77.333 | 0.000 |
| Winter | 6 | 249.371 | 78.287 | 0.000 |
| Winter * SumCum_Lag24 | 12 | 250.209 | 79.125 | 0.000 |
| Winter * SumCum_Lag21 | 12 | 252.029 | 80.945 | 0.000 |
| Sum7_Lag0 | 2 | 264.967 | 93.884 | 0.000 |
| SumCum_Lag21 | 2 | 338.672 | 167.588 | 0.000 |
| SumCum_Lag24 | 2 | 340.531 | 169.447 | 0.000 |
| NULL | 1 | 341.485 | 170.401 | 0.000 |
