# Supplementary material for: Cold-related Florida manatee mortality in relation to air and water temperatures
Source: PLoS One. 2019 Nov 21;14(11):e0225048. doi: 10.1371/journal.pone.0225048 (PMC6871784; doi:10.1371/journal.pone.0225048)
Supplement: S8 Table — Reports of cold-related carcasses were modeled using a Poisson generalized linear model, and models were ranked using the AICc value. Temperature variables used in the models are described in Fig 2. (DOCX) [file pone.0225048.s012.docx]

| Model | No. of  parameters | AICc | ΔAICc | Weight |
| --- | --- | --- | --- | --- |
| Winter + Sum14_Lag10 | 7 | 180.371 | 0.000 | 0.530 |
| Winter + Sum14_Lag10 + SumCum_Lag24 | 8 | 182.711 | 2.339 | 0.165 |
| Winter * Sum14_Lag10 | 12 | 183.242 | 2.871 | 0.126 |
| Winter + Sum14_Lag10 * SumCum_Lag24 | 9 | 183.567 | 3.196 | 0.107 |
| Winter + Sum7_Lag7 + Sum7_Lag14 | 8 | 187.086 | 6.715 | 0.018 |
| Winter + Sum7_Lag7 * Sum7_Lag14 | 9 | 188.782 | 8.411 | 0.008 |
| Winter + Sum7_Lag0 * Sum7_Lag14 | 9 | 188.897 | 8.526 | 0.007 |
| Winter + Sum7_Lag0 + Sum7_Lag7 + Sum7_Lag14 | 9 | 189.117 | 8.745 | 0.007 |
| Winter + Sum7_Lag14 | 7 | 189.209 | 8.838 | 0.006 |
| Winter + Sum7_Lag7 + Sum7_Lag14 + SumCum_Lag21 | 9 | 189.375 | 9.004 | 0.006 |
| Winter * Sum7_Lag14 | 12 | 189.943 | 9.572 | 0.004 |
| Winter + Sum7_Lag14 * SumCum_Lag21 | 9 | 190.732 | 10.361 | 0.003 |
| Winter + Sum14_Lag7 | 7 | 191.162 | 10.791 | 0.002 |
| Winter + Sum7_Lag14 + SumCum_Lag21 | 8 | 191.273 | 10.902 | 0.002 |
| Winter + Sum7_Lag0 + Sum7_Lag7 + Sum7_Lag14 + SumCum_Lag21 | 10 | 191.316 | 10.945 | 0.002 |
| Winter + Sum14_Lag7 + SumCum_Lag21 | 8 | 192.821 | 12.450 | 0.001 |
| Winter + Sum7_Lag0 + Sum14_Lag7 | 8 | 192.983 | 12.612 | 0.001 |
| Winter + Sum14_Lag7 * SumCum_Lag21 | 9 | 194.057 | 13.686 | 0.001 |
| Sum14_Lag10 * SumCum_Lag24 | 4 | 194.334 | 13.963 | 0.000 |
| Winter + Sum7_Lag0 * Sum14_Lag7 | 9 | 194.337 | 13.966 | 0.000 |
| Sum14_Lag10 | 2 | 194.630 | 14.258 | 0.000 |
| Winter + Sum7_Lag0 + Sum14_Lag7 + SumCum_Lag21 | 9 | 195.018 | 14.647 | 0.000 |
| Sum14_Lag10 + SumCum_Lag24 | 3 | 195.136 | 14.764 | 0.000 |
| Winter * Sum14_Lag7 | 12 | 195.662 | 15.290 | 0.000 |
| Sum7_Lag0 + Sum7_Lag7 + Sum7_Lag14 + SumCum_Lag21 | 5 | 198.480 | 18.108 | 0.000 |
| Sum7_Lag7 + Sum7_Lag14 + SumCum_Lag21 | 4 | 199.649 | 19.278 | 0.000 |
| Sum7_Lag0 + Sum7_Lag7 + Sum7_Lag14 | 4 | 200.340 | 19.968 | 0.000 |
| Sum7_Lag7 + Sum7_Lag14 | 3 | 201.272 | 20.901 | 0.000 |
| Sum14_Lag7 * SumCum_Lag21 | 4 | 201.422 | 21.050 | 0.000 |
| Sum7_Lag7 * Sum7_Lag14 | 4 | 201.597 | 21.226 | 0.000 |
| Sum14_Lag7 + SumCum_Lag21 | 3 | 202.351 | 21.980 | 0.000 |
| Sum7_Lag0 * Sum7_Lag14 | 4 | 203.384 | 23.013 | 0.000 |
| Sum7_Lag0 + Sum14_Lag7 + SumCum_Lag21 | 4 | 203.968 | 23.597 | 0.000 |
| Sum14_Lag7 | 2 | 205.583 | 25.212 | 0.000 |
| Sum7_Lag0 + Sum14_Lag7 | 3 | 207.563 | 27.192 | 0.000 |
| Sum7_Lag0 * Sum14_Lag7 | 4 | 207.696 | 27.325 | 0.000 |
| Sum7_Lag14 * SumCum_Lag21 | 4 | 210.079 | 29.708 | 0.000 |
| Sum7_Lag14 | 2 | 212.560 | 32.188 | 0.000 |
| Sum7_Lag14 + SumCum_Lag21 | 3 | 213.154 | 32.783 | 0.000 |
| Winter + Sum7_Lag0 * Sum7_Lag7 | 9 | 219.996 | 39.625 | 0.000 |
| Winter + Sum7_Lag7 | 7 | 225.614 | 45.243 | 0.000 |
| Winter + Sum7_Lag0 + Sum7_Lag7 | 8 | 227.077 | 46.706 | 0.000 |
| Winter + Sum7_Lag7 + SumCum_Lag21 | 8 | 227.956 | 47.584 | 0.000 |
| Winter + Sum7_Lag7 * SumCum_Lag21 | 9 | 229.572 | 49.201 | 0.000 |
| Winter * Sum7_Lag7 | 12 | 234.239 | 53.868 | 0.000 |
| Winter + SumCum_Lag24 | 7 | 249.173 | 68.802 | 0.000 |
| Winter + Sum7_Lag0 | 7 | 249.328 | 68.957 | 0.000 |
| Winter | 6 | 249.371 | 69.000 | 0.000 |
| Winter + SumCum_Lag21 | 8 | 250.287 | 69.916 | 0.000 |
| Winter * SumCum_Lag24 | 12 | 252.209 | 71.837 | 0.000 |
| Winter * SumCum_Lag21 | 12 | 253.846 | 73.475 | 0.000 |
| Sum7_Lag0 * Sum7_Lag7 | 4 | 254.537 | 74.166 | 0.000 |
| Winter * Sum7_Lag0 | 12 | 255.830 | 75.459 | 0.000 |
| Sum7_Lag7 * SumCum_Lag21 | 4 | 258.506 | 78.135 | 0.000 |
| Sum7_Lag7 + SumCum_Lag21 | 3 | 258.895 | 78.524 | 0.000 |
| Sum7_Lag7 | 2 | 263.522 | 83.151 | 0.000 |
| Sum7_Lag0 + Sum7_Lag7 | 3 | 265.023 | 84.652 | 0.000 |
| Sum7_Lag0 | 2 | 313.462 | 133.091 | 0.000 |
| SumCum_Lag21 | 2 | 338.929 | 158.558 | 0.000 |
| SumCum_Lag24 | 2 | 341.137 | 160.766 | 0.000 |
| NULL | 1 | 341.485 | 161.114 | 0.000 |
